# Update on Chitin and Chitosan from Insects: Sources, Production, Characterization, and Biomedical Applications

**DOI:** 10.3390/biomimetics9050297

**Published:** 2024-05-15

**Authors:** Zhenying Mei, Pavel Kuzhir, Guilhem Godeau

**Affiliations:** 1 Université Côte d’Azur, CNRS UMR 7010 Institut de Physique de Nice, 17 rue Julien Laupêtre, 06200 Nice, France; 2 Université Côte d’Azur, Institut Méditerranéen du Risque de l’Environnement et du Développement Durable, 9 rue Julien Laupêtre, 06200 Nice, France

**Keywords:** carbohydrate valorization, biopolymer, insect chitosan, extraction, characterization, industrial, biomedical, environmental applications

## Abstract

Insects, renowned for their abundant and renewable biomass, stand at the forefront of biomimicry-inspired research and offer promising alternatives for chitin and chitosan production considering mounting environmental concerns and the inherent limitations of conventional sources. This comprehensive review provides a meticulous exploration of the current state of insect-derived chitin and chitosan, focusing on their sources, production methods, characterization, physical and chemical properties, and emerging biomedical applications. Abundant insect sources of chitin and chitosan, from the Lepidoptera, Coleoptera, Orthoptera, Hymenoptera, Diptera, Hemiptera, Dictyoptera, Odonata, and Ephemeroptera orders, were comprehensively summarized. A variety of characterization techniques, including spectroscopy, chromatography, and microscopy, were used to reveal their physical and chemical properties like molecular weight, degree of deacetylation, and crystallinity, laying a solid foundation for their wide application, especially for the biomimetic design process. The examination of insect-derived chitin and chitosan extends into a wide realm of biomedical applications, highlighting their unique advantages in wound healing, tissue engineering, drug delivery, and antimicrobial therapies. Their intrinsic biocompatibility and antimicrobial properties position them as promising candidates for innovative solutions in diverse medical interventions.

## 1. Introduction

**Figure 1:**
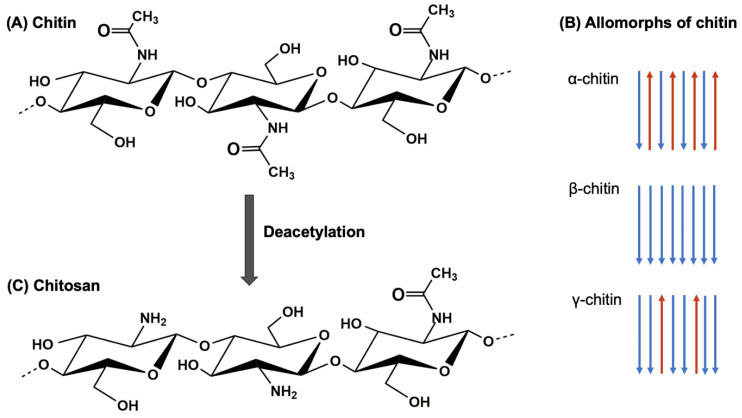
(**A**) Chitin molecular structure. (**B**) Three crystalline allomorphic forms of chitin. (**C**) Chitosan molecular structure.

Chitin, the second most abundant polysaccharide in nature after cellulose, composed of β-(1,4)-linked N-acetylglucosamine (GlcNAc) units, is a linear polymer that forms long chains with a crystalline structure (Figure 1A) [1]. This arrangement provides chitin with rigidity and insolubility in most solvents, underscoring chitin’s potential as a biomimetic archetype for materials characterized by exceptional mechanical attributes [2]. One notable aspect of chitin is the presence of three distinct allomorphs: α-chitin, β-chitin, and γ-chitin (Figure 1B) [3].

The first allomorph, α-chitin, is the most common and extensively studied. It features tightly antiparallel chains with intra- and intermolecular hydrogen bonding patterns. α-chitin exhibits a high degree of crystallinity, resulting in exceptional mechanical properties such as stiffness, tensile strength, and hardness [4]. It is commonly found in the exoskeletons of crustaceans and arthropods [5]. The second allomorph, β-chitin, possesses a more open and less ordered crystalline structure compared to α-chitin. It consists of parallel chains with weaker intermolecular hydrogen bonding, leading to reduced crystallinity. β-chitin exhibits lower mechanical strength but offers improved flexibility and less rigidity compared to α-chitin. It is present in the spines of diatoms, squid pens, and pogonophora tubes [6]. The least common and least studied allomorph is γ-chitin. It exhibits a helical arrangement of chains, resulting in a distinct hydrogen bonding pattern different from α-chitin and β-chitin [7]. γ-chitin is primarily found in fungi, yeasts, and insect cocoons [8,9,10]. The crystalline nature of chitin, along with the unique arrangements of the three allomorphs, influences mechanical strength, solubility, and degradation behavior. The hierarchical structure of chitin provides a model for the development of biomimetic materials with tailored mechanical properties. These include lightweight composites, protective coatings, and structural reinforcements. By mimicking the hierarchical organization and composition of chitin-rich structures found in nature, it is possible to engineer materials with superior strength-to-weight ratios, impact resistance, and self-healing capabilities.

Chitosan, a versatile polysaccharide mainly derived from the exoskeletons of crustaceans and insects, has garnered significant attention in recent years for its remarkable properties and potential biomedical applications [11]. Chitosan derived from chitin through deacetylation exhibits similar structural features with some modifications. The removal of acetyl groups introduces amino groups, altering the properties of the polymer (Figure 1C) [12]. The degree of deacetylation (DDA) determines the proportion of glucosamine units in the chitosan structure, influencing its solubility, charge density, and other physicochemical properties [13,14]. 

The structural characteristics of chitin and chitosan play a crucial role in determining their properties and applications. The crystalline nature of chitin, along with the unique arrangements of the three allomorphs, influences mechanical strength, solubility, and degradation behavior. Chitosan, with its improved solubility in acidic solutions and polycationic nature, exhibits enhanced bioactivity and interaction with biomolecules [15]. 

This transformation unlocks a myriad of opportunities for biomimetic exploration, enabling the development of materials that mimic biological tissues, extracellular matrices, and cellular environments. In biosensing and diagnostic applications, chitosan’s inherent biocompatibility and ability to interact with biomolecules make it an attractive candidate for the development of bioactive surfaces, biosensors, and diagnostic assays. The structural characteristics of chitin and chitosan play a crucial role in determining their properties and applications. These distinctive structural and property profiles of chitin and chitosan have made them highly valuable for biomedical applications. Their biodegradability, biocompatibility, and versatile functionalities make them attractive materials for drug delivery systems, tissue engineering, wound healing, and other therapeutic approaches. In particular, chitin and chitosan derived from insect sources have emerged as promising alternatives to traditional sources, offering a multitude of advantages over other origins [16]. 

This paper aims to provide an updated review of the advancements in chitin and chitosan from insects, focusing on its sources, production methods, characterization techniques, and biomedical applications, and highlighting the distinctive advantages it holds over other sources.

## 2. Sources of Chitin and Chitosan from Insects 

Insects present an abundant and sustainable source of chitin and chitosan, making them an attractive option for chitosan production. Unlike crustaceans, which are commonly utilized for chitosan extraction, insects offer several distinct advantages. 

On the one hand, insects have rapid reproduction rates, short lifecycles, and require minimal resources for cultivation, ensuring a consistent and readily available supply of chitin [17]. Chitin is distributed throughout an insect’s body, primarily in the exoskeleton but also present in other structures like the wings, antennae, and trachea [18]. This distribution allows for the extraction of chitin from various body parts, maximizing the utilization of insect biomass. An insect’s exoskeleton is composed mainly of chitin, providing structural support and protection for the insect’s body [19]. The use of insects as a raw material reduces the environmental impact associated with crustacean-based sources. This sustainability aspect aligns with the growing demand for environmentally friendly products.

Another advantage of insect-derived chitosan lies in its potential to reduce allergenicity compared to chitosan derived from crustaceans. Allergic reactions to chitosan from crustacean sources can occur due to the presence of allergenic proteins. However, insects possess different protein compositions, potentially lowering the risk of allergic reactions in individuals sensitive to crustaceans [20]. 

**Figure 2:**
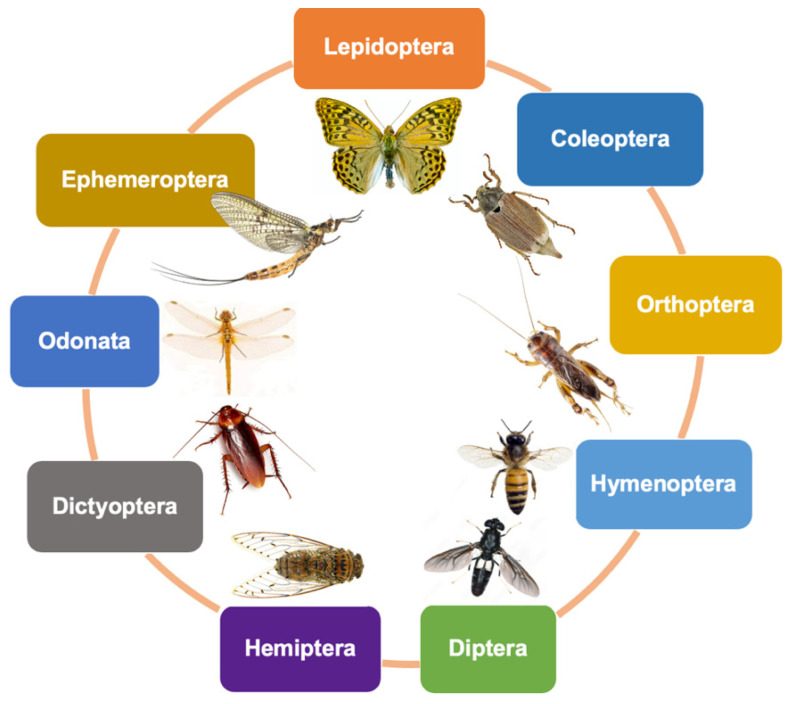
Insect sources of chitin and chitosan.

To date, few reviews have summarized the extraction, characterization, and biomedical applications of chitin and chitosan in insects. Here, a total of 82 insect species were collected, summarized, and analyzed (Table 1 and Figure 2).

**Table 1:** A summary of chemical methods for chitin purification from insects.

Insect Species	Demineralization	Deproteinization	Decoloration	Chitin Yield (%)	Ref.
**Lepidoptera**					
Silkworm	1 M HCl in 30 °C for 2 h	1 M NaOH in 90 °C for 2 h	2% KMnO_4_ for 2 h, 2% H_2_C_2_O_4_ for 2 h	NA	[21]
Flour moth, *Ephestia kuehniella*	1 M HCl at 100 °C for 20 min	1 M NaOH at 85 °C for 60 min	1% KMnO_4_ for 60 min	9.5–10.5	[22]
Butterfly, *Argynnis pandora*	2 M HCl at 50 °C for 24 h	2 M NaOH solution at 50 °C for 24 h	Distilled water, methanol, and chloroform (4:2:1) for 10 min	Wings—22 Body without wings—8	[23]
*Clanis bilineata*	7% (*v*/*v*) HCl at 25 °C for 24 h	10% (*w*/*v*) NaOH at 60 °C for 24 h	NA	NA	[24]
*Clanis bilineata*	7% (*v*/*v*) HCl at 25 °C for 24 h	10% (*w*/*v*) NaOH at 60 °C for 24 h	NA	NA	[25]
*Clanis bilineata* larvae	7% (*v*/*v*) HCl at 25 °C for 24 h	10% (*w*/*v*) NaOH at 60 °C for 24 h	NA	NA	[26]
**Coleoptera**					
Mealworm, *Tenebrio molitor*	1 M HCl in 30 °C for 2 h	1 M NaOH in 90 °C for 2 h	2% KMnO_4_ for 2 h, 2% H_2_C_2_O_4_ for 2 h	NA	[21]
Comb-clawed beetles, *Omophlus* sp.	2 M HCl for 4 h at 50 °C	2 M NaOH for 20 h at 100 °C	Methanol, chloroform, and water (2:1:4)	NA	[27]
Cockchafer, *Melolontha melolontha*	50 mL of 4 M HCl at 75 °C for 2 h	4 M NaOH at 150 °C for 18 h	Water, alcohol, and chloroform (4:2:1) for 20 min	13–14	[28]
Cockchafer, *Melolontha* sp.	2 M HCl at 60 °C for 20 h	1 M of NaOH for 20 h at 100 °C	Distilled water, methanol, and chloroform (4:2:1) for 30 min	Male—16.60 Female—15.66	[29]
Colorado potato beetle, *Leptinotarsa decemlineata*	100 mL of 2 M HCl at 65–75 °C for 2 h	50 mL of 2 M NaOH at 80–90 °C for 16 h	Chloroform, methanol, and water (in a ratio of 1:2:4) for 1 h	Adults—20 Larvae—7	[30]
*Catharsius molossus* L.	1.30 M HCl at 80 °C for 30 min	4.0 M NaOH at 90 °C for 6 h	2% oxalic acid at 70 °C for 30 min	24%	[31]
*Calosoma rugosa*	1 M HCl	1.0 M NaOH at 100 °C for 8 h	NA	5.0	[32]
*Calosoma rugosa*	* 36.5% HCl	* 1.0 M NaOH	NA	NA	[33]
Mealworm, *Tenebrio molitor*	3 h in 2 M HCl at 20 °C	500 mL 5% NaOH at 95 °C for 3 h	NA	18.01	[34]
Mealworm, *Tenebrio molitor*	2 M HCl at 50 °C for 24 h	2 M NaOH solution at 50 °C for 24 h	NA	NA	[35]
Mealworm, *Tenebrio molitor*	* 1.5 M HCl at 20 °C, 120 rpm for 6 h	* 1.25 M NaOH at 80 °C for 24 h	NA	4.72%	[36]
*Tenebrio Molitor*	2 M HCl at 65–75 °C for 2 h	2 M NaOH at 80 to 90 °C	Chloroform, methanol, and water (1:2:4) for 1 h	17.7%	[37]
Mealworm beetle, *Tenebrio molitor*, *Zophobas morio*	* 7% (*v*/*v*) HCl at 25 °C for 24 h	* NaOH at 80 °C for 24 h	NA	Larvae—4.60 Adult—8.40 Superworm—3.90	[38]
Rhinoceros beetle, *Allomyrina dichotoma*				Larvae—10.53 Pupa—12.70 Adult—14.20	
Mealworm, *Zophobas morio*	1.0 M HCl at 35 °C	0.5 M, 1.0 M and 2.0 M NaOH at 80 °C for 20 h	Glacial acetone for 30 min	0.5 M-5.43 1.0 M-5.22 2.0 M-4.77	[39]
Mealworm, *Zophobas morio*	20% HCI for 10 min	1 M NaOH 1:10 (g/mL) at 80 °C for 3 h	NA	NA	[40]
European stag beetle, *Lucanus cervus*	1 M HCl at 90 °C for 1 h	1 M NaOH in 90 °C for 14 h	Chloroform, methanol, and water (1:2:4, *v*/*v*)	10.9	[41]
Pine chafer, *Polyphylla fullo*				11.3	
*Pentodon algerinum*	5% acetic acid at 55 °C for 2 h	10% KOH at 40 °C for 48 h	NA	NA	[42]
Wheat weevil, *Sitophilus granarius*	1 M HCl for 0.5 h	1 M NaOH at 100 °C, 8 h	Ethanol and acetone	NA	[43]
Dor beetle, *Anoplotrupes stercorosus*	2 M of HCl at 100 °C for 2 h	2 M NaOH at 140 °C for 20 h	Chloroform, methanol, and water (1:2:4, *v*/*v*) for 2 h at room temperature	20.1	[44]
*Blaps tibialis*				25.2	
Rose chafer, *Cetonia aurata*				18.2	
Dor beetle, *Geotrupes stercorarius*				20.4	
*Blaps lethifera*	1 M HCl for 1 h at 40 °C	1 M NaOH at 80 °C for 2 h	10 *v*/*v* % H_2_O_2_ for 30 min at 50 °C	NA	[45]
*Pimelia fernandezlopezi*					
Banana weevil, *Cosmopolites sordidus*	1.0 M HCl at 50 °C for 24 h	1.0 M NaOH 80 °C for 8 h	NA	11.8	[46]
**Orthoptera**					
Grasshopper	1 M HCl in 30 °C for 2 h	1 M NaOH in 90 °C for 2 h	2% KMnO_4_ for 2 h, 2% H_2_C_2_O_4_ for 2 h	NA	[21]
*Shistocerca gregarea Forsskal*	5% acetic acid at 55 °C for 2 h	10% KOH at 40 °C for 48 h	NA	NA	[42]
Mexican katydid, *Pterophylla beltrani*	NA	NA	NA	11.8	[47]
Moroccan locust, *Dociostaurus maroccanus*	2 M HCl in 55 °C for 1 h	2 M NaOH in 50 °C for 18 h	Methanol, chloroform, and distilled water (2:1:4)	Nymphs—12 Adults—14	[48]
House cricket, *Brachytrupes*	* Oxalic acid for 3 h at room temperature	* 1 M NaOH at 95 °C for 6 h	1% sodium hypochlorite for 3 h	4.3–7.1	[49]
*Celes variabilis*,	4 M HCl at 75 °C for 2 h	4 M NaOH for 20 h at 150 °C	NA	4.71–11.84	[50]
Wart-biter, *Decticus verrucivorus*,					
Desert cricket, *Melanogryllus desertus*,					
*Paracyptera labiata*					
*Calliptamus barbarus * *Oedaleus decorus*	1 M HCl at 100 °C for 30 min	1 M NaOH at 80–90 °C for 21 h	Chloroform, methanol, and distilled water solution (1:2:4) for 1 h	20.5 16.5	[51]
*Ailopus simulatrix*	4 M HCl at 75 °C for 1 h	2 M NaOH at 175 °C for 18 h	Chloroform, methanol, and distilled water (1:2:4)	5.3	[52]
*Ailopus strepens*				7.4	
*Duroniella fracta*				5.7	
*Duroniella laticornis*				6.5	
Red-winged grasshopper, *Oedipoda miniata*				8.1	
Blue-winged grasshopper, *Oedipoda caerulescens*				8.9	
*Pyrgomorpha cognata*				6.6	
Two-spotted cricket, *Gryllus bimaculatus*	2 M HCl	1.25 M NaOH	NA	20.9–23.3	[53]
*Calosoma rugosa*	1 M HCl	1.0 M NaOH at 100 °C for 8 h	NA	12.2	[32]
*Bradyporus sureyai*	1 M HCl in 90 °C for 1 h	1 M NaOH in 90 °C for 14 h	Chloroform, methanol, and water (1:2:4, *v*/*v*)	9.8	[41]
European mole cricket, *Gryllotalpa gryllotalpa*				10.1	
Two-spotted cricket, *Gryllus bimaculatus*	* Oxalic acid for 3 h at room temperature	* 1 M NaOH at 95 °C and 130 rpm for 6 h	APS solution (50% (*w*/*v*)) at 50 °C for 30 min	5.1	[54]
Two-spotted cricket, *Gryllus bimaculatus*	2 M HCl at 21 °C for 3 h	1.25 M NaOH at 95 °C for 3 h	50% NaOH (*w*/*w*) at 95 °C and 105 °C for 3 h	79.03–91.14	[55]
*Brachystola magna*	2 M HCl at 50 °C for 24 h	2 M NaOH solution at 50 °C for 24 h	NA	NA	[35]
House cricket, *Acheta domesticus*	* 1 M HCl for 2 h at 98 °C	* 1 M NaOH at 80 °C for 24 h	NA	NA	[56]
House cricket, Acheta domesticus *Gryllodes sigillatus*	0.25 M HCl at 85 °C for 15 min	1 L NaOH at 70 °C for 22 h	NA	NA	[57]
House cricket, Acheta domesticus	* HCl, 1 M for 2 h at 98 °C	* NaOH, 1 M at 80 °C for 22 h	NA	7.34	[58]
House cricket, Acheta domesticus	1 M NaOH (1:2 *w*/*v*) at 70 °C for 22 h	0.25 M HCl (1:2 *w*/*v*) at 85 °C for 15 min	NA	5.7 ± 0.10	[59]
*Gryllodes sigillatus*				3.4 ± 0.10	
**Hymenoptera**					
Western honey bee, *Apsis mellifera*	2 M HCl at 80 °C for 6 h	2 M of NaOH and refluxed for 20 h at 100 °C	Distilled water (40 mL), methanol (20 mL), and chloroform (20 mL)	Head—8.9 Thorax—6.79 Abdomen—8.61 Legs—13.25 Wings—7.64	[60]
Western honey bee, *Apsis mellifera*	1 N HCl	1 M NaOH for 12 h at ambient temperature (20 °C)	NA	8.8	[61]
Western honey bee, *Apsis mellifera*	1 M HCl	1.0 M NaOH at 100 °C for 8 h	NA	2.5	[32]
Western honey bee, *Apsis mellifera*	36.5% HCl	1.0 M NaOH	NA	NA	[33]
Western honey bee, *Apis mellifera*	* 6.7% HCl at 25 °C for 3 h	* 8% NaOH at 90 °C for 1 h	33% H_2_O_2_	23	[62]
European hornet, *Vespa crabro* Oriental hornet, *Vespa orientalis* German wasp, *Vespula germanica*	2 M HCl at 75 °C for 2 h	4 M NaOH at 150 °C for 18 h	Distilled water, methanol, and chloroform (4:2:1) for 2 h	8.3 6.4 11.9	[63]
Asian hornet, *Vespa velutina*	100 mL of 1 M HCl at 50 °C for 3 h	1 M NaOH (100 mL) at 60 °C for 8 h	100 mL 1% sodium hypochlorite	11.7	[64]
Oriental hornet, *Vespa orientalis*	1.0 M HCl to a solid ratio of 15 mL/g at 100 °C for 20 min	1.0 M sodium hydroxide at 85 °C	H_2_O_2_/33% HCl 9:1, *v*/*v*	NA	[65]
European hornet, *Vespa crabro*	1 M HCl at 50 °C for 6 h	60 °C in 1 M NaOH solution for 16 h	Distilled water, methanol, and chloroform (4:2:1) solution at room temperature for 40 min at 250 rpm	Larvae—2.2 Pupa—6.2 Adult—10.3	[66]
Oriental hornet, *Vespa orientalis*	5% acetic acid at 55 °C for 2 h	10% KOH at 40 °C for 48 h	NA	NA	[42]
Red-tailed bumblebee, *Bombus lapidaries* *Formica clara*	2 M of HCl at 100 °C for 2 h	2 M NaOH at 140 °C for 20 h	Chloroform, methanol, and water (1:2:4, *v*/*v*) for 2 h at room temperature	9.3 7.8	[44]
**Diptera**					
Black soldier fly, *Hermetia illucens*	1:10 (*m*/*v*) with HCl 1 M at room temperature for 1 h	1 M NaOH treatment (solid: liquid ratio of 1:25 (*m*/*v*), 1 h at 80 °C	NA	Larvae—96.3 ± 3.7 Prepupae—94.5 ± 1.5 Pupae—93.9 ± 2.0 Shedding—75.7 ± 4.0 Cocoons—96.8 ± 1.8 Flies—95.7	[67]
Black soldier fly, *Hermetia illucens*	1 M HCl for 1 h	1 M NaOH at 80 °C for 24 h	1% KMnO_4_	NA	[68]
Black soldier fly, *Hermetia illucens*	1 M HCl at 100 °C for 30 min	1 M NaOH at 80 °C for 24 h	NA	Pupae exuviae—9 Imago—23	[69]
Black soldier fly, *Hermetia illucens*	NA	1 M NaOH 1 h at 80 °C	NA	8.5 ± 0.1	[70]
Black soldier fly, *Hermetia illucens*	2 M HCl at 55 °C for 1 h	2 M NaOH at 50 °C for 18 h	NaClO at 80 °C for 4 h	Larvae—3.6 Prepupa—3.1 Puparium—14.1 Adults—2.9	[71]
Black soldier fly, *Hermetia illucens*	HCl at 2 h	NaOH at 90 °C for 3 h	NA	21.3	[72]
Black soldier fly, *Hermetia illucens*	2% HCl for 2 h at 20 °C	NaOH 50 °C for 2 h	NA	7	[73]
Black soldier fly, *Hermetia illucens*	* 2 N HCl for 24 h at 15 min	* 40 mL of 2 N HCl for 24 h at room temperature	NA	9	[74]
Black soldier fly, *Hermetia illucens*	1 M HCl, 1:10 (*w*/*v*) for 2 h	1 M NaOH, 1 g/10 mL, at 80 °C for 6 h	1% KMnO_4_ in a 1:30 *w*/*v* ratio at room temperature for 4 h	Late larvae—3.025 Prepupae—5.371 Pupal exuviae—18.800 Imagoes—11.846	[75]
Black soldier fly, *Hermetia illucens*	* 1 M HCl for 2 h	* 1 M NaOH 4 h	NA	10.18 ± 0.42	[76]
Black soldier fly, *Hermetia illucens*	0.5 M CH_2_O_2_ for 1h at room temperature	2 M NaOH for 2 h at 80 °C	5% H_2_O_2_ for 1 h at 90 °C	NA	[77]
Black soldier fly, *Hermetia illucens*	1 M HCl for 2 h	1 M NaOH 4 h	NA	NA	[78]
Black soldier fly, *Hermetia illucens*	1 M HCl for 2 h at 100 °C	1 M NaOH for 4 h at 100 °C	NA	10.18	[79]
Black soldier fly, *Hermetia illucens*	0.5 M formic acid for 1 h at room temperature	2 M NaOH, 2 h at 80 °C	5% (*v*/*v*) H_2_O_2_ for 30–60 min at 90 °C	Larvae—10 ± 0.7 Pupal exuviae—23 ± 1.9 Dead adults—6 ± 0.1	[80]
Black soldier fly, *Hermetia illucens*	0.5 M HCl at room temperature for 2 h	1.9 M NaOH for 2 h at 50 °C	5% H_2_O_2_	NA	[81]
Black soldier fly, *Hermetia illucens*	7% HCl for 2 h at room temperature	10% NaOH at 80 °C for 24 h	NA	NA	[82]
Black soldier fly, *Hermetia illucens*	1% HCl at 20 °C for 2 h	30% (*w*/*w*) NaOH at room temperature for 30 min, and then at 100 °C for 2 h	NA	NA	[83]
Black soldier fly, *Hermetia illucens*	1 M HCl at 22 °C for 1 h	1 M NaOH at 80 °C for 24 h	1—Without decoloration 2—Water at 100 °C for 24 h 3—9% H_2_O_2_ at 80 °C for 2.5 h 4—9% H_2_O_2_ at 80 °C for 5 h 5—1% KMnO_4_ at 80 °C for 20 min	1—7.95 ± 0.20 2—7.97 ± 0.10 3—7.01 ± 0.12 4—5.98 ± 0.08 5—5.69 ± 0.28	[84]
Common fruit fly *Drosophila melanogaster*	2 M HCl solution for 3 h at 4 °C	NaOH (8% *w*/*w*) solution for 20 h at 70 °C	Methanol, chloroform, and distilled water (in a ratio of 2:1:4) for 30 min	7.85	[85]
*Calliphora vicina*	2 M of HCl at 100 °C for 2 h	2 M NaOH at 140 °C for 20 h	Chloroform, methanol, and water (1:2:4, *v*/*v*) for 2 h at room temperature	8.1	[44]
Housefly, *Musca domestica*	1 M HCl for 1 h at 40 °C	2 h of 1 M NaOH at 80 °C	10 *v*/*v* % H_2_O_2_ for 30 at 50 °C	NA	[45]
Housefly, *Musca domestica*	3 h in 500 mL of 2 N HCl solution at room temperature	500 mL of 1.25 N NaOH at 95 °C for 3 h	NA	8.02	[86]
*Tabanus bovinus*	1 M HCl for 12 h at room temperature	1 M NaOH for 18 h at 70 °C	Water, methanol, and chloroform (1:2:4)	NA	[87]
**Hemiptera**					
Green bug, *Nezara viridula*	5% acetic acid at 55 °C for 2 h	10% KOH at 40 °C for 48 h	NA	NA	[42]
Dock bug, *Coreus marginatus*	2 M of HCl at 100 °C for 2 h	2 M NaOH at 140 °C for 20 h	Chloroform, methanol, and water (1:2:4, *v*/*v*) for 2 h at room temperature	14.5	[44]
Black-and-red bug, *Lygaeus equestris*				11.1	
*Pyrrhocoris apterus*				10.6	
Cicada slough	1 M HCl in 30 °C for 2 h	1 M NaOH in 90 °C for 2 h	2% KMnO_4_ for 2 h, 2% H_2_C_2_O_4_ for 2 h	NA	[21]
Aquatic bug, *Ranatra linearis*	100 mL of 1 M HCl at 90 °C for 1 h	1 M NaOH at 110 °C for 18 h	Chloroform, methanol, and water (1:2:4)	15–16	[88]
*Cicada lodosi*	2 M HCl for 2 h at 100 °C	2 M NaOH at 100 °C for 20 h	Water, methanol, and chloroform mixed at a ratio of 4:2:1	4.97	[89]
*Cicada mordoganensis*				6.49	
*Cicadatra platyptera*				8.84	
*Cicadatra atra*				6.70	
*Cicadatra hyaline*				5.51	
*Cicadivetta tibialis*				5.88	
*Cicada Cryptotympana atrata*	1000 mL of 7% (*w*/*w*) HCl at room temperature for 24 h	1000 mL of 10% (*w*/*w*) NaOH at 60 °C for 24 h	NA	62.42	[24]
*Coridius nepalensis*	1 M HCl for 1 h	1 M NaOH at 80 °C for 24 h	1% sodium hypochlorite for 1 h	43.97	[90]
**Dictyoptera**					
*Eupolyphaga sinensis*	1.3 M HCl at 80 °C for 1 h, soaked at room temperature for 24 h	4 M NaOH at 90 °C for 6 h	10% H_2_O_2_ at 80 °C for 30 min	11.63 ± 0.80	[91]
Brazilian cockroach, *Blaberus giganteus*	NA	2 M NaOH at 90 °C for 9 h	Chloroform, methanol, and water (1:2:2) at room temperature for 1.5 h	Wings—26.9 Dorsal pronotum—21.2	[92]
German cockroach, *Blattela germanic*	5% acetic acid at 55 °C for 2 h	10% KOH at 40 °C for 48 h	NA	NA	[42]
German cockroach, *Blattella germanica*	2 M of HCl at 100 °C for 2 h	2 M NaOH at 140 °C for 20 h	Chloroform, methanol, and water (1:2:4, *v*/*v*) for 2 h at room temperature	4.7	[44]
American cockroach, *Periplaneta americana*	1% sodium hypochlorite solution (1%, *w*/*v*)	1 M NaOH at 100 °C for 24 h	NA	Nymph—8.4 Adult—15	[93]
German cockroach, *Blattella germanica*				Nymph—5.4 Adult—6.2	
American cockroach, *Periplaneta americana*	2 N HCl at room temperature for 3 h	1.25 N NaOH at 95 °C for 3 h	NA	3.36	[53]
American cockroach, *Periplaneta americana*	* 20 mL of 1% HCl for 24 h	* 4% of NaOH for 1 h	50 mL of 2% NaOH for 1 h	NA	[94]
American cockroach, *Periplaneta americana*	4 M HCl for 2 h at 75 °C	4 M NaOH for 20 h at 150 °C	Water, methanol, and chloroform (ratio of 4:2:1) for 4 h at 30 °C	Wings—18 Without wings—13	[95]
American cockroach, *Periplaneta americana*	* 0.5 M HCl at 60 °C and 500 rpm for 1 h	* 0.5 M NaOH at 95 °C and 500 rpm for 20 min, then 4 M NaOH for 160 min	10% H_2_O_2_ at 80 °C and 500 rpm for 3 h	NA	[96]
American cockroach, *Pariplaneta americana linnaeus*	6.7% HCl at 25 °C for 3 h	8% NaOH at 90 °C for 1 h	33% H_2_O_2_	42	[62]
American cockroach, *Periplaneta americana*	* 1 M HCl for 2 h at 75 °C	* 2.5% (*w*/*v*) NaOH for 6 h at 100 °C	Acetone at 50 °C for 2 h	NA	[97]
American cockroach, *Periplaneta americana*	5% acetic acid at 55 °C for 2 h	10% KOH at 40 °C for 48 h	NA	NA	[42]
American cockroach, *Periplaneta americana*	NA	4% NaOH for 48 h at 90 °C	NA	NA	[98]
**Odonata**					
Dragonfly, *Sympetrum fonscolombii*	1 M HCl at room temperature for 1 h	1 M NaOH solution at 50 °C for 15 h	Chloroform, methanol, and distilled water (1:2:4, *v*/*v*)	20.3 ± 0.85	[99]
Downy emerald, *Cordulia aenea*	2 M of HCl at 100 °C for 2 h	2 M NaOH at 140 °C for 20 h	Chloroform, methanol, and water (1:2:4, *v*/*v*) for 2 h at room temperature	9.5	[44]
Four-spotted chaser, *Libellula quadrimaculata*				10.1	
**Ephemeroptera**					
Mayfly	2 M HCl at 50 °C	2 M NaOH at 100 °C	Methanol and chloroform (1:1)	10.21	[100]

* In this literature, the deproteinization step is performed prior to demineralization. NA: not available. Highlights: abundant insect sources of chitin from the Lepidoptera, Coleoptera, Orthoptera, Hymenoptera, Diptera, Hemiptera, Dictyoptera, Odonata, and Ephemeroptera orders; hydrochloric acid (HCl) is effective in demineralizing chitin, facilitating the removal of calcium compounds and other minerals; sodium hydroxide (NaOH) and potassium hydroxide (KOH) are commonly used for deproteinization, effectively removing protein residues from the chitin matrix; some methods employ bleaching agents to remove pigments and enhance chitin purity; chitin yields vary depending on the insect species and the purification method employed, ranging from 3.3% to 96.8%.

**Table 2:** A summary of methods for chitin deacetylation.

Insect Species	Deacetylation Conditions	Chitosan Yield (%)	DDA (%)	Molecular Weight (Da)	Moisture Content (%)		Ash Content (%)		Ref.
					Chitin	Chitosan	Chitin	Chitosan	
**Lepidoptera**									
Silkworm	60% NaOH in 100 °C for 8 h	3.1	85.5	(4.090 ± 0.059) × 10^4^	NA	0.07 ± 0.008	NA	0.05 ± 0.003	[20]
Mediterranean flour moth, *Ephestia kuehniella*	NA	NA	NA	NA	9.1 ± 0.4	NA	0.14 ± 0.08	NA	[21]
*Clanis bilineata*	55% NaOH (*w*/*w*), 120 °C for 4 h	95.9	NA	NA	NA	3.8	NA	0.3	[24]
**Coleoptera**									
Mealworm, *Tenebrio molitor*	60% NaOH in 100 °C for 8 h	2.5	85.9	(3.975 ± 0.072) × 10^4^	NA	0.19 ± 0.012	NA	0.50 ± 0.016	[20]
Mealworm, *Tenebrio molitor*	500 mL of NaOH at 95 or 105 °C for 3 h or 5 h	9.2	95.5	NA					[33]
Mealworm, *Tenebrio molitor*	50% NaOH at 100 °C for 3h	78.26	75.84	NA					[36]
Mealworm, *Tenebrio molitor*	60% NaOH contained NaBH_4_ (0.004 g), 120 °C for 2 h	NA	88.55	8.123×10^5^	NA				[33]
Mealworm, *Tenebrio molitor*	NaOH 40% (*w*/*v*) at 90 °C, 500 rpm for 8 h	31.9	53.9	NA	6.2 ± 0.5	4.2 ± 0.1	3.6 ± 0.2	3.7 ± 0.1	[101]
Mealworm, *Tenebrio molitor*	50% NaOH at 80 °C for 4 h	NA	89.4	NA					[35]
Mealworm beetle, *Tenebrio molitor*, *Zophobas morio*	55% (*w*/*v*) NaOH at 90 °C for 9 h	Larvae—80.00 Adult—78.33 Superworm—83.33	Larvae—75.59 Adult—75.63 Superworm—75.67	NA					[37]
Rhinoceros beetle, *Allomyrina dichotoma*		Larvae—83.37 Pupa—83.37 Adult—75.00	Larvae—75.66 Pupa—75.67 Adult—74.66						
Mealworm beetle, *Zophobas morio*	50 wt % NaOH in 90 °C for 30 h	65.84, 70.88, 75.52	81.06, 64.82, 74.14	NA					[39]
Mealworm beetle, *Zophobas morio*	NaOH 100 g/mL at 80 °C for 16 h	15–18%	NA	NA					[40]
*Catharsius molossus*	8 M NaOH at room temperature for 24 h	17%	94.9 ± 0.85	4.5 ± 0.07 ×10^5^	NA	6.55 ± 0.05	NA	0.34 ± 0.04	[31]
*Calosoma rugosa*	50% NaOH (15 mL/g) at 100 °C for 8 h	NA	95	NA	NA	8.8	NA	2.0	[32]
*Calosoma rugosa*	50% NaOH at 100 °C for 8 h	NA	95	NA					[33]
Colorado potato beetle, *Leptinotarsa decemlineata*	50% NaOH (*w*/*v*, 1:20) at 100 °C for 3 h	Adult—72 Larvae—67	Adult—82 Larvae—76	Adults—2.722 × 10^3^ Larvae—2.676 × 10^3^	NA				[102]
European stag beetle, *Lucanus cervus*,	NA	NA	NA	NA	6.6	NA	0.6	NA	[41]
Pine chafer, *Polyphylla fullo*					5.9		1.7		
*Blaps lethifera*,	50 *w*/*v*% NaOH	50.0 ± 0.3	87.1 ± 0.2	NA	NA	14.3 ± 0.3	NA	1.5 ± 0.1	[45]
*Pimelia fernandezlopezi*		41.7 ± 0.5	88.2 ± 0.1			17.2 ± 0.2		2.0 ± 0.1	
Banana weevil, *Cosmopolites sordidus*	50% NaOH at 90 °C for 10 h	70.2	77.8 ± 0.39	(343 ± 37.3) × 10^3^	NA	2.4	6.4	2.2	[46]
**Orthoptera**									
Grasshopper	60% NaOH in 100 °C for 8 h	5.7	89.7	(3.989 ± 0.021) × 10^4^	NA	1.8 ± 0.213	NA	0.89 ± 0.025	[21]
Mexican katydid, *Pterophylla beltrani*	70% NaOH in 1:3 ratio for 1.5 h at 120 °C	58.8	NA	NA	NA	NA	NA	NA	[46]
Moroccan locust, *Dociostaurus maroccanus*	60% NaOH at 150 °C for 4 h	Nymphs—77.38 Adults—81.69	NA	Adults—7.2 × 10^3^ Nymphs—5.6 × 10^3^	NA	NA	NA	NA	[48]
*Brachytrupes*	50% (*w*/*v*) NaOH at 121 °C for 5 h	2.4–5.8	NA	NA	4.00	3.33	1.00	1.00	[49]
*Calliptamus barbarus*, *Oedaleus decorus*	50% NaOH (*w*/*v* 1:15) at 130 °C for 2 h	70–75 74–76	70–75	NA	NA	NA	NA	NA	[51]
Desert locust, *Schistocerca gregaria*	50% NaOH (15 mL/g) at 100 °C for 8 h	NA	98	NA	NA	NA	14.1	1.6	[32]
*Bradyporus sureyai*,	NA	NA	NA	NA	5.2	NA	3.8	NA	[41]
European mole cricket, *Gryllotalpa gryllotalpa*					6.0		2.1		
Two-spotted cricket, *Gryllus bimaculatus*	(50–67% NaOH) at 95 °C at 130 rpm	41.75	56.47–84.98	NA	NA	NA	NA	NA	[54]
*Brachystola magna*	60% NaOH contained NaBH_4_ (0.004 g), 120 °C for 2 h	NA	89.89 ± 1.34	696.95 × 10^3^	NA	NA	NA	NA	[35]
House crickets, *Acheta domesticus*	50% NaOH for 3 h at 130 °C	88.0	62.9	86.8 × 10^3^	NA	NA	NA	NA	[56]
House crickets, *Acheta domesticus*	40% NaOH at 120 °C for 2 h	90.6	88.5	NA	NA	NA	NA	NA	[103]
House crickets, *Acheta domesticus*	67% *w*/*v* NaOH for 2, 4 6, 10 h	2 h-76.0 ± 6.7 4 h-77.3 ± 1.9 6 h-80.5 ± 2.1 10 h-69.0 ± 2.2	2 h-72.5 ± 1.0 4 h-76.3 ± 1.3 6 h-79.1 ± 1.9 10 h-79.4 ± 1.3	344 × 10^3^	NA	NA	NA	NA	[59]
*Gryllodes sigillatus*		2 h-65.0 ± 1.6 4 h-63.7 ± 1.2 6 h-60.3 ± 3.3 10 h-62.3 ± 0.9	2 h-73.5 ± 1.4 4 h-74.9 ± 1.3 6 h-77.2 ± 1.8 10 h-81.3 ± 1.1	524 × 10^3^					
**Hymenoptera**									
Western honey bee, *Apsis mellifera*	NA	NA	NA	NA	7.7 ± 0.09	NA	2.4 ± 0.03	NA	[61]
Western honey bee, *Apsis mellifera*	50% NaOH at 100 °C for 8 h	NA	96	NA	NA	17.6	NA	9.2	[32]
Oriental hornet, *Vespa orientalis*	50% NaOH at 100 °C for 2 h	NA	96	NA	NA	NA	NA	NA	[65]
**Diptera**									
Black soldier fly, *Hermetia illucens*	NaOH at 100 °C for 2 h	32	90	NA	NA	NA	NA	NA	[73]
Black soldier fly, *Hermetia illucens*	50% (*w*/*v*) NaOH (1: 50) for 4 h at 95 °C	Late larvae—81.034 Prepupae—73.656 Pupal exuviae—79.701 Imagoes—63.158	NA	NA	NA	NA	NA	NA	[75]
Black soldier fly, *Hermetia illucens*	40% NaOH for 8 h	6.58	NA	NA	NA	NA	NA	NA	[76]
Black soldier fly, *Hermetia illucens*	12 M NaOH for 4 h at 100 °C	Larvae—3 Pupal exuviae—10 Dead adults—3	Larvae—92 Pupal exuviae—90 Dead adults—93	Larvae—21 × 10^3^ Pupal exuviae—35 × 10^3^ Dead adults—36 × 10^3^	NA	NA	NA	NA	[80]
Black soldier fly, *Hermetia illucens*	100 °C for 2 h	81	66	505 × 10^3^	NA	NA	NA	NA	[83]
Common fruit fly, *Drosophila melanogaster*	10 mL of NaOH solution (60%, *w*/*w*) for 48 h at 150 °C	70.91	NA	NA	NA	NA	NA	NA	[85]
Oriental blue fly, *Chrysomya megacephala*	100 mL NaOH (1 mol/L) at 95 °C for 6 h	26.2	89.6	501 × 10^3^	NA	NA	NA	NA	[104]
Housefly, *Musca domestica*	50% NaOH	57.9 ± 0.2	84.1 ± 0.3	NA	NA	7.8 ± 0.1	NA	8.2 ± 0.2	[45]
**Hemiptera**									
Cicada slough	60% NaOH at 100 °C for 8 h	28.2	84.1	(3.779 ± 0.068) × 10^4^	NA	0.18 ± 0.016	NA	0.03 ± 0.004	[21]
Aquatic bug, *Ranatra linearis*	NA	70%	NA	NA	NA	NA	NA	NA	[88]
**Dictyoptera**									
American cockroach, *Periplaneta americana*	50% NaOH in 100 °C for 4 h	5.80	36.8	NA	NA	NA	NA	NA	[105]
German cockroach, *Blattella germanica*		2.95	31.5						
American cockroach, *Periplaneta americana*	40% NaOH in 120 °C for 2 h	99.7	90.3	NA	NA	NA	NA	NA	[103]
American cockroach, *Periplaneta americana*	10% NaOH at 80 °C for 3 h	NA	90.85 ± 3.37	(16 ± 0.746) × 10^3^	NA	NA	NA	NA	[96]
American cockroach, *Periplaneta americana*	50% (*w*/*v*) NaOH for 8 h at 120 °C	74.51	NA	NA	NA	NA	NA	NA	[97]
*Eupolyphaga sinensis*	50% NaOH at 90 °C for 24 h	5.48 ± 0.32	96.57 ± 0.48	(127.79 ± 1.35) × 10^3^	NA	5.19 ± 0.11	NA	0.55 ± 0.05	[91]
**Ephemeroptera**									
Mayfly	60% NaOH at 150 °C for 6 h	78.43	84.3	3.69 × 10^3^	NA	NA	NA	NA	[100]

NA: not available. Highlights: abundant insect sources of chitin from the Lepidoptera, Coleoptera, Orthoptera, Hymenoptera, Diptera, Hemiptera, Dictyoptera, and Ephemeroptera orders; sodium hydroxide (NaOH) is commonly used for deacetylation; yield, degree of deacetylation (DDA), and molecular weight (Mw) of chitosan vary depending on the insect species and the purification method employed, ranging from 2.5% to 99.7%, 31.5% to 96.57%, and 2.676 × 10^3^ Da to 8.123 × 10^5^ Da, respectively; moisture content and ash content of chitin and chitosan ranged from 4.0% to 9.1% and 0.07% to 17.6%, respectively; ash content of chitin and chitosan ranged from 0.14% to 14.1% and 0.03% to 9.2%, respectively.

**Table 3:** Elemental analysis (EA) results of chitin and chitosan from insects.

Insect Species	Chitin (%)				Chitosan (%)				Ref.
	Carbon (C)	Hydrogen (H)	Nitrogen (N)	Carbon/Nitrogen	Carbon (C)	Hydrogen (H)	Nitrogen (N)	Carbon/Nitrogen	
Flour moth, *Ephestia kuehniella*	43.12	6.11	5.89	7.057	NA	NA	NA	NA	[22]
Butterfly, wings, *Argynnis pandora*	44.89	6.53	6.62	6.781	NA	NA	NA	NA	[23]
Butterfly, OBP, *Argynnis pandora*	44.91	6.45	6.48	6.931	NA	NA	NA	NA	
Common cockchafer, *Melolontha melolontha*	45.09	6.29	6.72	6.71	NA	NA	NA	NA	[28]
Superworm, *Zophobas morio*	0.5 M-43.27 1.0 M-43.07 2.0 M-43.32	0.5 M-6.77 1.0 M-6.73 2.0 M-6.77	0.5 M-6.60 1.0 M-6.38 2.0 M-6.29	0.5 M-6.56 1.0 M-6.75 2.0 M-6.89	42.76 42.27 42.08	7.47 7.09 7.40	6.55 6.76 6.56	6.53 6.25 6.41	[39]
European stag beetle, *Lucanus cervus*	45.9	7.6	5.3	8.5	NA	NA	NA	NA	[41]
Pine chafer, *Polyphylla fullo*	45.4	7.5	5.1	8.9					
Moroccan locust, *Dociostaurus maroccanus*	Adult—42.35 Nymph—47.32	Adult—5.64 Nymph—6.64	Adult—4.63 Nymph—5.66	Adult—9.15 Nymph—8.36	Adult—41.63 Nymph—42.01	Adult—6.38 Nymph—6.28	Adult—7.20 Nymph—6.45	Adult—5.78 Nymph—6.51	[48]
House cricket, *Brachytrupes*	41.30	NA	6.022	6.858	38.98	NA	5.932	6.571	[49]
*Celes variabilis*	45.05–49.0	6.31–6.92	5.68–6.43	7.01–8.24	NA	NA	NA	NA	[50]
Wart-biter, *Decticus verrucivorus*									
*Melanogryllus desertus*									
*Paracyptera labiata*									
*Bradyporus sureyai* European mole cricket, *Gryllotalpa gryllotalpa*	46.6 ± 0.1 44.2 ± 0.1	7.7 ± 0.1 7.6 ± 0.1	5.3 ± 0.1 5.0 ± 0.1	8.8 8.8	NA	NA	NA	NA	[41]
European hornet, *Vespa crabro*	46.62	6.42	6.85	6.81	NA	NA	NA	NA	[63]
Oriental hornet, *Vespa orientalis*	46.01	6.34	6.71	6.86					
German wasp, *Vespula germanica*	44.94	5.95	6.90	6.51					
Asian hornet, *Vespa velutina*	43.47	6.94	6.85	6.35	NA	NA	NA	NA	[64]
European hornet, *Vespa crabro*	Larval—45.6 Pupa—46.2 Adult—45.5	Larval—6.5 Pupa—6.4 Adult—6.3	Larval—6.5 Pupa—6.3 Adult—6.49	Larval—7.02 Pupa—7.33 Adult—7.01	NA	NA	NA	NA	[66]
Black soldier fly, *Hermetia illucens*	Pupal exuviae—35.23 Imago—32.09	Pupal exuviae—5.11 Imago—4.80	Pupal exuviae—3.73 Imago—3.9	Pupal exuviae—9.45 Imago—8.23	NA	NA	NA	NA	[68]
Black soldier fly, *Hermetia illucens*	Pupae exuviae—43.74 Imago—39.74	Pupae exuviae—5.82 Imago—5.46	Pupae exuviae—6.14 Imago—6.00	Pupae exuviae—6.62 Imago—7.12	NA	NA	NA	NA	[69]
Black soldier fly, *Hermetia illucens*	41.84	6.74	5.96	7.02	NA	NA	NA	NA	[73]
Oriental blue fly, *Chrysomya megacephala*	NA	NA	NA	NA	39.06 ± 0.20	7.16 ± 0.08	7.30 ± 0.08	5.35	[104]
American cockroach, *Periplaneta americana*	45.74	6.59	6.69	6.84	NA	NA	NA	NA	[95]
Dragonfly, *Sympetrum fonscolombii*	47.09	6.65	6.83	6.89	NA	NA	NA	NA	[99]
American cockroach, *Pariplaneta Americana linnaeus*	47.23	7.32	7.20	6.56	NA	NA	NA	NA	[62]
Western honey bee, *Apis mellifera linneaus*	52.65	8.42	5.55	9.49	NA	NA	NA	NA	[62]
*Coridius nepalensis*	42.175	6.551	6.878	6.13	NA	NA	NA	NA	[90]
American cockroach, *Periplaneta americana*	43.84	6.93	6.33	6.92	NA	NA	NA	NA	[98]
German cockroach, *Blattella germanica*	46.28	6.84	6.84	6.77	NA	NA	NA	NA	[44]
Dor beetle, *Anoplotrupes stercorosus*	40.6	7.66	6.35	6.39					
*Blaps tibialis*	45.17	6.85	6.43	7.02					
Rose chafer, *Cetonia aurata*	43.6	7.13	6.87	6.35					
*Geotrupes stercorariu*	43.1	6.49	6.77	6.37					
*Calliphora vicina*	48.9	6.54	6.79	7.2					
Dock bug, *Coreus marginatus*	39.2	6.95	6.03	6.5					
Black-and-red bug, *Lygaeus equestris*	46.59	6.34	6.74	6.91					
*Pyrrhocoris apterus*	46.38	6.02	6.77	6.85					
Red-tailed bumblebee, *Bombus lapidaries*	40.1	7.48	6.11	6.56					
*Formica clara*	46.48	6.45	6.59	7.05					
Downy emerald, *Cordulia aenea*	44.6	6.86	6.66	6.70					
Four-spotted chaser, *Libellula quadrimaculata*	43.0	6.95	6.42	6.70					
Pale giant horse-fly, *Tabanus bovinus*	47.60	6.55	6.57	7.24	41.99	6.42	7.18	5.85	[87]

NA: not available. Highlights: the percentage of C atoms from chitin and chitosan originating from various insects ranged from 32.09 to 48.90% and 38.98 to 42.76%, respectively; the N value of chitin and chitosan from various insects ranged from 4.63 to 6.9% and 5.93 to 7.3%, respectively; the carbon/nitrogen (C/N) ratio of chitin and chitosan for various insects ranged from 6.13 to 9.49 and 5.35 to 6.57, respectively.

**Table 4:** XRD peaks and crystalline index value (%) of chitin and chitosan from insects.

Insect Species	Chitin (%)		Chitosan (%)		Ref.
	XRD Peaks at 2θ	CrI (%)	XRD Peaks at 2θ	CrI (%)	
**Lepidoptera**					
Silkworm	9.6, 19.7, 12.7, 23.2, 26.3, 39	59.21	10, 20	32.9	[21]
Butterfly, *Argynnis pandora*	Wings—9.3, 9.3, 12.84, 21.04, 22.9, 26.36 OBP—8.5, 19.3, 12.84, 21.14, 23.06, 6.66	Wings—64 OBP—66	NA NA	NA NA	[23]
**Coleoptera**					
Mealworm	9.6, 19.7, 12.7, 23.2, 26.3, 28.1, 39	81.11	10, 20	51.9	[21]
*Omophlus* sp.	9.42, 12.72, 19.34, 20.84, 23.32, 26.44	82.9	NA	NA	[27]
Cockchafer, *Melolontha melolontha*	NA	75.2	NA	NA	[28]
Cockchafer, *Melolontha* sp.	9.32–9.70, 12.12–13.22, 12.78–13.22, 19.18–19.76, 20.06–21.48, 23.06–23.78, 26.02–26.80	74.1–88.9	NA	NA	[29]
Colorado potato beetle, *Leptinotarsa decemlineata*	Larvae—9.6,13.22, 19.68, 21.42, 23.26, 26.7 Adults—9.66, 13.18, 19.48, 21.06, 23.16, 26.76	Larvae—72 Adults—76	NA	NA	[30]
*Calosoma rugosa*	NA	NA	9.7, 20.3, 22.6	49	[32]
*Calosoma rugosa*	NA	NA	9.7, 20.3	49	[33]
Mealworm beetle, *Tenebrio molitor*	9.6, 19.6, 21.1, 23.7, 36	57.85	NA	NA	[36]
Mealworm beetle, *Tenebrio molitor*	9.011, 9.034, 19.119, 19.167, 21.38, 21.44, 22.68, 22.74	NA	NA	NA	[106]
Mealworm beetle, *(Tenebrio molitor*, *Zophobas morio)*	NA	NA	10.62, 20.02	58.11	[38]
Rhinoceros beetle, *Allomyrina dichotoma*	NA	NA	10.74, 19.92	62.77	
European stag beetle, *Lucanus cervus*	9.67, 12.40, 19.60, 23.41, 26.26, 39.1	85.2	NA	NA	[41]
Pine chafer, *Polyphylla fullo*	9.2, 12.40, 19.46, 23.50, 26.21, 28.1, 39.5	86.1			
Wheat weevil, *Sitophilus granarius*	8.9, 9.2, 18.7, 25.6, 12.3, 22.8	78.77	NA	NA	[43]
Dor beetle, *Anoplotrupes stercorosus*	9.58, 13.36, 19.66, 21.14, 23.18, 26.52	83.5	NA	NA	[44]
*Blaps tibialis*	9.48, 12.76, 19.38, 21.08, 23.04, 26.64	80.1			
Rose chafer, *Cetonia aurata*	9.44, 13.04, 19.52, 21.28, 23.46, 26	86.3			
*Geotrupes stercorarius*	9.64, 13.14, 19.56, 21.38, 23.22, 26.76	80.1			
*Blaps lethifera*	NA	NA	10.7, 19.9	84	[45]
*Pimelia fernandezlopezi*			11.5, 20.4	73	
Banana weevil, *Cosmopolites sordidus*	NA	NA	19.5	41	[46]
**Orthoptera**					
Grasshopper	9.6, 19.7, 12.7, 23.2, 26.3, 28.1, 39.0	83.4	10, 20	50.1	[21]
Moroccan locust, *Dociostaurus maroccanus*	Adult—9.56, 12.76, 19.72, 21.12, 23.96, 26.64 Nymph—9.42, 12.86, 19.72, 21.56, 23.38, 26.66	Adult—71 Nymph—74	Adult—10.96, 20.14 Nymph—10.76, 20.3	NA	[48]
House cricket, *Brachytrupes*	9.4, 12.8, 17.1, 19.4, 21.1, 23.2, 26.3, 28.5, 35.0, 39.0	88.02	9.6, 19.6, 21.2	86.64	[49]
*Celes variabilis*	9, 19, 12, 21, 23, 26	75–80	NA	NA	[50]
Wart-biter, *Decticus verrucivorus*					
*Melanogryllus desertus*					
*Paracyptera labiata*					
*Calliptamus barbarus*	9.26, 19.28, 21.24, 23.28, 26.36, 31.78	70.9	10.92, 20.08	NA	[51]
*Oedaleus decorus*	9.6, 19.6, 21.1,23.7, 26.64	76.8	10.08, 20.14		
*Ailopus simulatrix*	9.3, 12.7, 19.6, 21.1, 23.8, 26.6	76	NA	NA	[52]
*Ailopus strepens*	9.5, 12.8, 19.6, 20.8, 23.8, 26.4	75			
*Duroniella fracta*	9.5, 12.6, 19.4, 20.9, 23.5, 26.8	72			
*Duroniella laticornis*	9.5, 12.8, 19.3, 20.7, 23.2, 26.5	71			
Red-winged grasshopper, *Oedipoda miniata*	9.7, 12.9, 19.6, 21, 23.7, 26,8	74			
*Oedipoda caerulescens*	9.3, 12.7, 19.3, 20.7, 23.1, 26.9	74			
*Pyrgomorpha cognata*	9.4, 13.3, 19.6, 20.9, 23.4, 26,9	63			
Desert locust, *Schistocerca gregaria*	NA	NA	9.3, 20.2, 24.4	69	[32]
*Bradyporus sureyai*	9.62, 12.5, 19.72, 23.74, 26.22, 27.8, 39.2	83.1	NA	NA	[41]
European mole cricket, *Gryllotalpa gryllotalpa*	9.44, 12.3, 19.41, 23.31, 26.2, 27.9, 39.0	80.6			
Two-spotted cricket, *Gryllus bimaculatus*	NA	NA	10.50, 20.07	57.52	[54]
*Shistocerca gregarea Forsskal*	9.2, 19.1,12.6, 22.9, 26.2	71.4	NA	NA	[42]
**Hymenoptera**					
Western honey bee, *Apsis mellifera*	NA	NA	9.7, 20.3	59	[32]
Western honey bee, *Apsis mellifera*	NA	NA	9.7, 20.3	59	[33]
European hornet, *Vespa crabro*	9.64, 12.74, 19.38, 20.94, 23.92, 26.88	69.88	NA	NA	[63]
Oriental hornet, *Vespa orientalis*	9.68, 12.72, 19.32, 21.6, 23.74, 26.8	53.92			
European wasp, *Vespula germanica*	9.32, 12.92, 20.l04, 21.24, 23.16, 25.9	50			
Oriental hornet, *Vespa orientalis*	9.2, 19.1,12.6, 22.9, 26.2	39.4	NA	NA	[42]
Red-tailed bumblebee, *Bombus lapidarius*	9.64, 13.02, 19.58, 21.22, 23.44, 26.78	75.5	NA	NA	[44]
*Formica clara*	9.5, 13.38, 19.78, 20.84, 23.1, 26.76	69.8			
**Diptera**					
Musca domestica	NA	NA	10.5, 20.2	81	[45]
Black soldier fly, *Hermetia illucens*	9.4, 13.0, 19.3, 20.8, 23.2, 29.5	Prepupae—94 Cocoons—94 Sheddings—89 Larvae—89	NA	NA	[67]
Black soldier fly, *Hermetia illucens*	9, 19, 22, 24, 30	Larval—35 Imago—24.9	NA	NA	[68]
Black soldier fly, *Hermetia illucens*	9.3, 19.8, 23, 26.0	Pupae exuvia—25.2 Imago—49.4	NA	NA	[69]
Black soldier fly, *Hermetia illucens*	Larvae—9.30, 12.78, 19.26, 21.82, 23.31, 26.41 Prepupa—9.38, 12.93, 19.33, 21.19, 23.42, 26.37 Puparium—9.30, 12.67, 19.29, 20.77, 23.38, 26.45 Adult—9.50, 12.82, 19.33, 20.81, 23.31, 26.34	Larvae—33.09 Prepupa—35.14 Puparium—68.44 Adult—87.92	NA	NA	[71]
Black soldier fly, *Hermetia illucens*	9, 19, 13, 21, 23, 26	Larvae—84 Pupal exuviae—62 Dead adults—93	10, 20	Larvae—77 Pupal exuviae—80 Dead adults—86	[80]
*Calliphora vicina*	9.38, 12.88, 19.3, 20.8, 22.84, 26.8	67.1	NA	NA	[44]
**Hemiptera**					
Cicada slough	9.6, 19.7, 12.7, 23.2, 26.3, 39	85.21	10, 20, 23.2	49.1	[21]
*Aquatic bug*, *Ranatra linearis*	9.34, 12.38, 19.66, 20.88, 23.22, 26.56, 38.96	84.8	NA	NA	[88]
*Coridius nepalensis*	9, 20, 20.5, 22.8, 26.4	86.33	NA	NA	[90]
Dock bug, *Coreus marginatus*	9.7, 13.2, 19.86, 21.24, 23.42, 26.54	76.9	NA	NA	[44]
Black-and-red bug, *Lygaeus equestris*	9.64, 13, 19.76, 21.16, 22.8, 26.7	59.7			
*Pyrrhocoris apterus*	9.44, 12.52, 19.14, 20.84, 22.66, 26.7	62.1			
**Dictyoptera**					
American cockroach, *Periplaneta americana*	9.14, 19.58, 12.88, 20.98, 23.12, 26.8	86.7	NA	NA	[95]
American cockroach, *Periplaneta americana*	12, 19, 20.5, 21.5, 23, 26	83.72	NA	NA	[98]
German cockroach, *Blattela germanica*	9.2, 19.1, 12.6, 22.9, 26.2	44.2	NA	NA	[42]
German cockroach, *Blattella germanica*	9.4, 12.7, 19.5, 20.68, 23.33, 26.66	70.1	NA	NA	[44]
**Odonata**					
Dragonfly, *Sympetrum*, *fonscolombii*	9, 13, 19, 21, 26	96.4	NA	NA	[99]
Downy emerald, Cordulia aenea	9.54, 13.18, 19.62, 21.4, 23.76, 26.92	73.2	NA	NA	[44]
Four-spotted chaser, Libellula quadrimaculata	9.54, 13.24, 19.68, 21.06, 23.1, 26.88	63.9			

NA: not available. The crystallinity of chitin and chitosan from different insect species ranged from 24.9 to 96.4% and 32.9% to 86.64%, respectively; the typical peaks at 2θ for chitin are around 9–10°, 13°, 20–22°, 23°, and 26°. The typical peaks at 2θ for chitosan are around 10° and 20–25°.

**Table 5:** Thermogravimetric analysis (TGA) of insect chitin and chitosan.

Insect Species	Chitin (%)					Chitosan (%)					Ref.
	First		Second		DTG_max_ (°C)	First Peak		Second Peak		DTG_max_ (°C)	
	Mass Loss (%)	T (°C)	Mass Loss (%)	T (°C)		Mass Loss (%)	T (°C)	Mass Loss (%)	T (°C)		
**Lepidoptera**											
Silkworm, *Bombyx mori*	NA	NA	NA	NA	NA	NA	80–90	NA	290–300	NA	[21]
Butterfly, *Argynnis pandora*	Wings—4.85 OBP—4.82	30–100	Wings—82.23 OBP—80	100–650	Wings—386.9 OBP—399.6	NA	NA	NA	NA	NA	[23]
**Coleoptera**											
Mealworm, *Tenebrio molitor*	NA					NA	80–90	NA	290–300	NA	[21]
*Omophlus* sp.	3.6% (α) 7.7% (β)	NA	78.8% (α) 63.5% (β)	NA	385.3 (α) 334.2 (β)	NA	NA	NA	NA	NA	[27]
Cockchafer, *Melolontha melolontha*	4	0–150	78	150–600	380	NA	NA	NA	NA	NA	[28]
Cockchafer, *Melolontha* sp.	1.1–5.4	NA	51.2–94.5	NA	Female—392.2 Male—378.3	NA	NA	NA	NA	NA	[29]
Colorado potato beetle, *Leptinotarsa decemlineata*	3–5	NA	Adult—74 Larval—48	NA	Adult—379 Larval—307	3–5	NA	94–96	NA	Adult—289 Larval—292	[102]
*Catharsius molossus*	NA					13.37	25–290	45.76	290–400	317.70	[31]
European stag beetle, *Lucanus cervus*	6.6	100	70	300–420	379.9	NA	NA	NA	NA	NA	[41]
Pine chafer, *Polyphylla fullo*	5.9	100	73		374.7						
Dor beetle, *Anoplotrupes stercorosus*	4	NA	74	NA	387	NA	NA	NA	NA	NA	[44]
*Blaps tibialis*	7		76		385						
Rose chafer, *Cetonia aurata*	7		82		361						
*Geotrupes stercorarius*	5		77		390						
**Orthoptera**											
Grasshopper	NA					NA	80–90	NA	290–300	NA	[21]
Moroccan locust, *Dociostaurus maroccanus*	4	NA	Adult—77 Nymph—82	NA	Adult—386 Nymph—383	Adult—5 Nymph—7	NA	Adult—62 Nymph—59	NA	Adult—302 Nymph—308	[48]
*Celes variabilis*	3–6	0–150	73–96	150–400	350–387	NA	NA	NA	NA	NA	[50]
Wart-biter, *Decticus verrucivorus*											
*Melanogryllus desertus*											
*Paracyptera labiata*											
*Calliptamus barbarus*	8	0–150	72	150–650	381	8	0–150	61	300	296	[51]
*Oedaleus decorus*	6		77		390	6		57		305	
*Ailopus simulatrix*	NA	0–150	82	150–600	383	NA	NA	NA	NA	NA	[52]
*Ailopus strepens*			78		382						
*Duroniella fracta*			74		381						
*Duroniella laticornis*			72		382						
Red-winged grasshopper, *Oedipoda miniata*			76		385						
*Oedipoda caerulescens*			77		384						
*Pyrgomorpha cognata*			74		384						
*Bradyporus sureyai*	5.2	0–100	72	300–420	382.4	NA	NA	NA	NA	NA	[41]
European mole cricket, *Gryllotalpa gryllotalpa*	6.0		70	300–412	374.6						
House crickets, *Acheta domesticus*	7.4	0–150	53	200–400	359.2	NA	NA	NA	NA	NA	[56]
**Hymenoptera**											
Western honey bee, *Apsis mellifera*	Head—6 Thorax—4 Abdomen—3 Legs—5 Wings—3	NA	Head—67 Thorax—56 Abdomen—68 Legs—68 Wings—60	NA	Head—308 Thorax—360 Abdomen—367 Legs—359 Wings—359	NA	NA	NA	NA	NA	[60]
European hornet, *Vespa crabro*	Larvae—3.51 Pupa—2.7 Adult—6.5	30–180	Larvae—88.7 Pupa—69.9 Adult—78.3	NA	Larvae—384.8 Pupa—381.7 Adult—382.4	NA	NA	NA	NA	NA	[66]
Red-tailed bumblebee, *Bombus lapidarius*	5	NA	72	NA	384	NA	NA	NA	NA	NA	[44]
*Formica clara*	4		78		374						
**Diptera**											
Black soldier fly, *Hermetia illucens*	2–3	0–122	62–63	122–450	Imago—387 Larval—389	NA	NA	NA	NA	NA	[68]
Black soldier fly, *Hermetia illucens*	5–6	74–110	70–80	250	Imago—363 Pupae exuvia—371	NA	NA	NA	NA	NA	[69]
Black soldier fly, *Hermetia illucens*	Larvae—4.42 Prepupa—6.74 Puparium—8.52 Adults—7.5	0–150	Larvae—69.48 Prepupa—71.16 Puparium—71.25 Adults—3.31	150–400	Larvae—372 Prepupa—373 Puparium—371 Adults—372	NA	NA	NA	NA	NA	[71]
Common fruit fly, *Drosophila melanogaster*	4.4	NA	75.6	NA	378.7	4.47	NA	61.12	NA	304.7	[85]
*Calliphora vicina*	4	NA	65	NA	379	NA	NA	NA	NA	NA	[44]
Pale giant horse-fly, *Tabanus bovinus*	4.6	30–150	80.2	200–650	370.50	7.3	30–150	59.3	200–650	295.4	[87]
**Hemiptera**											
Cicada slough	NA					NA	80–90	NA	290–300	NA	[21]
*Aquatic bug*, *Ranatra linearis*	6	0–150	78	150–650	393	9	0–150	50	150–650	289	[88]
*Cicada lodosi*	4.41	0–200	83.94	200–750	411.70	NA	NA	NA	NA	NA	[89]
*Cicada mordoganensis*	4.88		80.44		412.40						
*Cicadatra platyptera*	3.80		81.78		412.20						
*Cicadatra atra*	4.54		83.75		411.50						
*Cicadatra hyaline*	5.4		66.78		412.70						
*Cicadivetta tibialis*	4.04		73.49		339.90–402.30						
Dock bug, *Coreus marginatus* Black-and-red bug, *Lygaeus equestris* *Pyrrhocoris apterus*	9 3 5	NA	73 66 78	NA	389 375 387	NA	NA	NA	NA	NA	[44]
**Dictyoptera**											
American cockroach, *Periplaneta americana*	5	100	76	350–390	389	NA	NA	NA	NA	NA	[95]
German cockroach, *Blattella germanica*	4	NA	77	NA	389	NA	NA	NA	NA	NA	[44]
**Odonata**											
Dragonfly, *Sympetrum fonscolombii*	2.9	25–100	73.2	100–750	369.2	NA	NA	NA	NA	NA	[99]
Downy emerald, *Cordulia aenea*	4	NA	75	NA	378	NA	NA	NA	NA	NA	[44]
Four-spotted chaser, *Libellula quadrimaculata*	6		76		384						

NA: not available. The typical DTG_max_ of chitin and chitosan extracted from different insect orders ranged between 307 and 412.7 °C and 289 and 317.7 °C, respectively.

**Table 6:** ^13^C NMR spectral data of chitin and chitosan in different insect sources.

Insect Species	Chemical Shift (ppm)								Ref.
	C1	C2	C3	C4	C5	C6	C=O	CH_3_	
Blowfly larvae, chitosan	104.47	56.78	75.14	85.31	75.14	60.41	NA	22.64	[59]
Black soldier fly, chitin	104.6	55.7	74.2	84.0	76.4	61.5	173.9	23.4	[69]
European rhinoceros beetle, *Oryctes nasicornis*, chitin film	104	55	73	83	75	61	NA	NA	[107]
Asian hornet, *Vespa velutina*, chitin	104.12	55.94	NA	82.74	75.34	60.34	173.17	22.79	[64]
Black soldier fly, *Hermetia illucens* Pupal, chitin	104.3	55.2	73.5	83.2	75.8	60.9	173.1	22.9	[108]
Mealworm, *Tenebrio molitor* Linnaeus 1758, chitin	104.63	55.71	76.14	83.63	76.14	61.46	173	23	[109]
Monarchs, Swallowtails, chitin	104	NA	73	83	76	NA	173	23	[110]

NA: not available.

## 3. Extraction of Chitin and Chitosan from Insects

**Figure 3:**
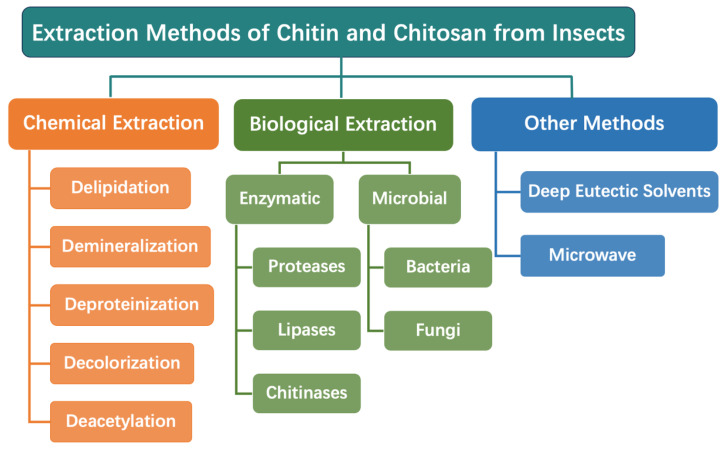
Production process for chitin and chitosan from insects.

The production process of insect-derived chitin and chitosan entails a multi-step approach typically involving delipidation, deproteination, demineralization, and deacetylation (Figure 3). Various extraction techniques, such as chemical, enzymatic, and microbial methods, have been developed to efficiently extract chitosan from insect sources. These techniques aim to maximize chitosan yield while ensuring the preservation of its structural integrity and desired properties.

### 3.1. Chemical Extraction

The sequence of the processes involved in the extraction of chitin and chitosan may vary depending on the type of insect source (Table 1 and Table 2). Regarding delipidation, some literature performs it in the first step and some literature in the last step. In most of the literature, the demineralization step is performed prior to deproteinization. In some of the literature, the deproteinization step is performed prior to demineralization. The results after the statistics showed that out of 80 articles related to insect chitin extraction, only 13 of them were deproteinized first and then demineralized. Decolorization usually follows demineralization and deproteinization.

#### 3.1.1. Delipidation

Delipidation methods for insects can vary significantly across different studies and applications (Table 1). Chloroform is a commonly used solvent for delipidation. It is highly effective in extracting lipids, but its high toxicity requires proper safety precautions. Methanol is a safer alternative compared to chloroform, and it is often used alone or as part of a solvent mixture. Chloroform–methanol is a widely used solvent mixture for delipidation. It is a popular choice for its high lipid solubility and relatively low toxicity compared to pure chloroform. Adelya et al. used 500 mL of chloroform–methanol (7:3) mixture to treat 100 g of larval shells at 20 °C for 4 h, and the yield of chitin-containing material (defatted material) was 93% [73]. Tsaneva et al. extracted the lipid fraction from honeybees (*Apis mellifera*) with n-hexane in a Soxhlet apparatus for 8 h before demineralization and deproteinization. The wax content in the lipid fraction was determined gravimetrically as 24.9 ± 0.28% [61]. Ethanol is also used for delipidation of insect samples, especially in food-related studies [62,111]. It is relatively safe and can be an appropriate choice for certain applications. Honeybee (*Apis mellifera*) was also defatted with 96% ethanol in Soxhlet’s extraction apparatus for 18 h [62]. Kaya et al. removed lipids from honeybee (*Apis mellifera*) using a mixture of distilled water (40 mL), methanol (20 mL), and chloroform (20 mL), stirring for 40 min at 250 rpm after demineralization and deproteinization [60]. In another study, for the defatting of chitin isolation, Kaya et al. used chloroform–methanol–water (1:2:4, *v*:*v*) and refluxed for 2 h at room temperature [44]. Son et al. removed lipids from mealworms with n-hexane in a shaker at 170 rpm for 6 h [36]. *Eupolyphaga sinensis* Walker insects were defatted with 95% ethanol (*m*/*v*, 1/5) and petroleum ether (*m*/*v*, 1/3) at 65 °C for 2 h each time [91]. *Periplaneta americana* was defatted using hexane as the solvent with a Soxhlet extraction method in a water bath at 80 °C for 3 h [96]. In some studies, other solvents like a mixture of acetone and alcohol or petroleum ether have been used for delipidation [74,112].

#### 3.1.2. Demineralization 

Demineralization methods for insects involve the removal of mineral components, primarily calcium carbonate, from the exoskeleton to isolate chitin and chitosan. Acetic acid and hydrochloric acid are the most used reagents for demineralization (Table 1). Studies have shown that both acids can effectively remove calcium carbonate from insect exoskeletons [21,42]. Acetic acid is generally considered safer to handle compared to hydrochloric acid. However, using hydrochloric acid might lead to faster and more complete demineralization in some cases. Both acetic acid and hydrochloric acid do not significantly affect the chitin and chitosan structure. They mainly act on the mineral component, leaving the chitin and chitosan intact. However, prolonged exposure to strong acids or elevated temperatures might lead to some degree of depolymerization of chitin and chitosan, reducing their molecular weight and viscosity. In some studies, other acids like sulfuric, nitric, oxalic, and formic acids have been used for demineralization [9,49,80,113]. Except for solvents, the choice of duration and temperature in demineralization can significantly affect the efficiency of the process and properties of the extracted chitin and chitosan. In determining the optimal demineralization conditions, the specific characteristics of the insect species as well as the intended applications of the extracted chitin and chitosan need to be considered. 

#### 3.1.3. Deproteinization 

Deproteinization methods for insects involve removing proteins and organic matter to isolate chitin and chitosan. The solvent concentration in deproteinization is crucial, as it influences protein removal efficiency and the properties of the extracted chitin and chitosan. The choice of concentration should consider both deproteinization efficiency and the impact on chitin and chitosan properties. Sodium hydroxide (NaOH) is the most used deproteinization agent (Table 1). Studies have used varying concentrations of NaOH, typically ranging from 1 M to 4 M [21,23,28]. While less commonly used than NaOH, potassium hydroxide (KOH) was employed for deproteinization in some studies [42,114]. High temperatures (70–100 °C) are commonly used for deproteinization [22,28,34]. This helps improve deproteinization efficiency, especially for more resistant proteins. However, higher temperatures may increase the risk of chitin and chitosan degradation and must be carefully controlled. Optimizing regeant concentration and temperature in deproteinization methods according to the specific insect species and desired properties of extracted chitin and chitosan is essential for efficient protein removal while preserving quality. This is crucial for diverse applications, including industrial, biomedical, or environmental uses.

#### 3.1.4. Decolorization 

Decolorization methods for insects involve the removal of pigments and other color-causing compounds from the insect material to obtain decolored chitin and chitosan. Sodium hypochlorite, potassium permanganate, hydrogen peroxide, and chloroform–methanol–water (1:2:4, *v*/*v*) are commonly used as bleaching agents for decolorization (Table 1) [21,44,45,64]. In some studies, acidic solutions such as oxalic acid or hydrochloric acid have been used for decolorization [31,64]. Acidic conditions can help in breaking down pigments, but care must be taken to prevent excessive degradation of chitin and chitosan. The choice of solvents, duration, and temperature in decolorization can significantly impact the efficiency of the process and the properties of the resulting chitin and chitosan. Room temperature (20–25 °C) is a common temperature range for decolorization [44,64]. Room temperature decolorization generally minimizes chitin and chitosan degradation. Mild heating (40–100 °C) can enhance decolorization efficiency, especially for more resistant pigments [31,45].

#### 3.1.5. Deacetylation

Deacetylation methods for insects involve the removal of acetyl groups from chitin to produce chitosan. NaOH is the most used solvent for deacetylation (Table 2). Concentrations of NaOH ranging from 40% to 70% have often been used in different studies [21,47,97,103]. Deacetylation conditions can vary depending on the insect species and DDA. The concentration of these alkalis, duration, and temperature should be optimized for efficient deacetylation while preserving the integrity of chitosan. A temperature range of 80–120 °C is common for deacetylation [25,53,91,105].

### 3.2. Biological Extraction

Biological extraction methods for chitin and chitosan from insects involve the use of enzymatic microbial processes to break down the insect material and release the desired biopolymers. These methods offer a more environmentally friendly and sustainable approach compared to chemical extraction methods.

Enzymatic extraction involves the use of specific enzymes that can selectively degrade the non-chitin components of the insect material, leaving behind chitin and chitosan. Enzymes such as proteases, lipases, and chitinases are used to break down proteins, lipids, and chitin–protein complexes in the insect material. Andressa et al. used Alcalase enzyme in a proportion of 2% (*w*/*w*; enzyme/substrate) for the deproteinization of mealworm cuticles [101]. The enzymatic extraction process is typically carried out under controlled conditions, including optimized pH and temperature, to ensure the effectiveness of the enzymes. Enzymatic extraction offers the advantage of specific targeting, minimal damage to chitin and chitosan structures, and reduced chemical waste.

Microbial extraction involves the use of microorganisms, such as bacteria or fungi, that naturally produce enzymes capable of degrading insect material. Microbes can be cultivated in a suitable growth medium containing the insect material, allowing them to release enzymes that break down proteins, lipids, and other organic matter. Yun et al. used purified protease from *Bacillus licheniformis* and *Bacillus subtilis* for deproteinization and defatting of black soldier fly exoskeletons, respectively [115]. Lin et al. used a *Bacillus licheniformis* A6 strain in the fermentation of spent pupal shells of black soldier flies for chitin extraction [116]. Marios et al. isolated chitin from house crickets (*Acheta domesticus*) by comparing microwave-assisted demineralization to a chemical method, fermentation with Lactococcus lactis, and citric acid treatment, leading to a degree of demineralization of 85.8 ± 1.3%, 91.1 ± 0.3%, 97.3 ± 0.8%, and 70.5 ± 3.5%, respectively [56]. The microbial fermentation process is typically carried out under controlled conditions to promote the growth and activity of the microorganisms. The resulting mixture is then processed to separate and isolate the chitin and chitosan from the microbial biomass and other residual components.

Biological extraction methods offer a sustainable and eco-friendly approach by minimizing the use of harsh chemicals and reducing chemical waste. These methods, milder in nature, help preserve the inherent properties of chitin and chitosan structures. While scalable for industrial production, challenges include the need for optimization due to factors like enzyme or microbial choice, process conditions, and insect species. Despite their environmental benefits, biological extraction methods may entail additional steps, such as enzyme production or microbial cultivation, potentially increasing overall production costs compared to chemical methods. Scaling up these methods to meet industrial demands may pose challenges in terms of process control, scalability, and cost-effectiveness.

### 3.3. Other Extraction Methods

In recent years, several alternative methods have been suggested to replace the chemical treatments in chitin isolation and chitosan preparation, including deep eutectic solvents (DES) and microwave extraction. Ionic liquids are liquid salts composed of ions, typically consisting of an organic cation and an inorganic or organic anion. Ionic liquids have shown promise in the extraction of chitin and chitosan from insect exoskeletons due to their ability to dissolve and solvate biomolecules effectively [117]. They allow for liberating chitin with simultaneous removal of the protein–mineral matrix, without the need for prior isolation and purification of chitin polymer. This process reduces the amount of required chemicals and decreases the volume of process waste while avoiding the necessity of handling boiling acidic solutions used in the traditional preparation process [118]. Furthermore, ionic liquids can be tailored by choosing specific cations and anions to optimize their interactions with target molecules, thus enhancing the efficiency of the extraction process [119]. DES is a subclass of ionic liquids formed by the combination of hydrogen bond acceptors and donors. Unlike traditional ionic liquids, DES are typically composed of inexpensive and biodegradable components, making them environmentally friendly alternatives [120]. Their ability to disrupt hydrogen bonds in the insect exoskeleton facilitates the dissolution of chitin, enabling efficient extraction. Gaël et al. compared the impact of both DES/IL pretreatments on the efficiency of the chemical deacetylation of chitin carried out over two insect sources (*Bombyx eri* and *Hermetia illucens*) and shrimp shells, resulting in chitosan obtained from IL-pretreated chitins from *Bombyx eri* larva presenting lower acetylation degrees (13–17%) than DES-pretreated samples (18–27%) [121].

Furthermore, microwave-assisted extraction is a modern and efficient technique for extracting chitin and chitosan from insect exoskeletons. This method utilizes microwave energy to accelerate the extraction process, leading to faster and higher yields compared to traditional extraction methods. Microwave extraction relies on the principle of selective heating. When exposed to microwave radiation, polar molecules like water and certain solvents absorb the energy and convert it into heat. This localized and rapid heating promotes the breakdown of cell walls and enhances the diffusion of the solvent into the insect exoskeleton, facilitating the extraction of chitin and chitosan [122]. Leke et al. compared the extraction of chitin from black soldier fly (*Hermetia illucens*) meal using conventional and alternative methods: enzyme-, microwave-, and ultrasound-assisted extraction. The results showed that the conventional method resulted in 9.7% chitin yield, while the enzyme-, microwave-, and ultrasound-assisted extractions yielded 42.3%, 11.4%, and 13.7% chitin on dry weight basis, respectively [14].

## 4. Characterization and Modification of Chitin and Chitosan from Insects

The characterization of insect-derived chitin and chitosan plays a pivotal role in evaluating its quality and suitability for diverse biomedical applications. Employing an array of analytical techniques, including spectroscopy, microscopy, thermal analysis, and rheology, enables the assessment of its physicochemical properties involving molecular weight distribution, degree of deacetylation, and other pertinent characteristics. The resulting characterization data furnish invaluable insights into the structure–function relationships of insect chitosan, thus facilitating tailored modification of its properties to suit specific applications.

### 4.1. Physicochemical and Structural Characterization

#### 4.1.1. Extraction Yield

The yield of chitin and chitosan from insects refers to the quantitative measure of these biopolymers obtained after the extraction process. It is a critical parameter that assesses the efficiency of the extraction method and the potential feasibility of using insects as a viable source for chitin and chitosan production. Typically expressed as a percentage or weight relative to the initial mass of the insect exoskeleton, the yield is an essential determinant of the success of chitin and chitosan extraction. The yield of chitin and chitosan from insects is shown in Table 1 and Table 2. Numerous factors influence the yield of chitin and chitosan from insects. Different insect species exhibit varying chitin and chitosan contents in their exoskeletons. Variations in chitin content among insect species can result in significant differences in yield. The chitin yield extracted from various Coleoptera insects was found to vary in a wide range, such as *Tenebrio molitor* (3.9–4.6%), *Melolontha melolontha* (13–14%), *Leptinotarsa decemlineata* (7–20%), *Heliocopris dilloni* (22.1%), *Catharsius molossus* L. (24%), *Blaps tibialis* (25%), and *Mecynorhina torquata* (27%), respectively [28,31,38,44,45,102,123,124]. The chitin yield extracted from various Orthoptera insects, such as *Gryllodes sigillatus*, *Brachytrupes*, *Pterophylla beltrani*, *Oedaleus decorus*, and *Gryllus bimaculatus*, was found to be 3.4%, 4.3–7.1%, 11.8%, 16.5%, and 79.03–91.14%, respectively [47,49,51,55,59]. The chitosan yield extracted from various Lepidoptera insects was found to vary widely, such as silkworm chrysalis (3.1%) and *Clanis bilineata* (95.9%), respectively [2,10]. The contents of chitin and chitosan can vary with age and molting stage of insects. Different life cycle stages may lead to fluctuations in overall yield. The yields of chitin from *Leptinotarsa decemlineata* varied between adult (20%) and larvae (7%) [30]. The yields of chitin from *Hermetia illucens* varied between late larvae (3.025%), prepupae (5.371%), pupal exuviae (18.800%), and imagoes (11.846%), respectively [75]. Various experimental parameters, including extraction time, temperature, solvent concentration, and solid–liquid ratio, play pivotal roles in determining the yield. Many studies have been reported on extracting chitin and chitosan from *Hermetia illucens*, and the chitin and chitosan content after the deproteinization, demineralization, and decoloration processes varied greatly, between 3.1 and 96.3% and 3 and 81% [11,12,13,14,15,16,17,18,19,20,21,22,23,24,25,26,27]. Finding and selecting new sources of chitin and chitosan that can replace crustacean shells and optimize the yield is particularly important for industrial applications, as this directly affects the cost-effectiveness of the extraction process.

#### 4.1.2. Degree of Deacetylation (DDA)

As described in Section 2, the DDA of chitosan refers to the extent of acetyl group removal from the chitin polymer, resulting in the formation of chitosan. Chitosan is a linear polysaccharide composed of β-(1→4)-linked D-glucosamine (GlcN) and N-acetyl-D-glucosamine (GlcNAc) units. The deacetylation process involves the hydrolysis of GlcNAc units to GlcN units, and the DDA is expressed as a percentage representing the proportion of GlcN units in the chitosan chain. The DDA of chitosan from insects is a crucial parameter as it directly influences physicochemical and biological properties, making it suitable for a wide range of applications in various industries. 

The DDA of chitosan from insects can be determined using various analytical methods, such as nuclear magnetic resonance (NMR), Fourier Transform Infrared Spectroscopy (FTIR), the potentiometric titration method, and the acid–base titration method. It is essential to control and optimize the deacetylation process to achieve chitosan with the desired DDA for specific applications. The DDA of chitosan from insects is shown in Table 2. It was reported that the DDA of chitosan of Lepidoptera insects like silkworms was 85.5% [21]. The DDA of chitosan of Coleoptera insects like *Tenebrio molitor*, *Zophobas morio*, *Allomyrina dichotoma*, *Catharsius molossus*, *Calosoma rugosa*, *Leptinotarsa decemlineata*, *Blaps lethifera*, *Pimelia fernandezlopezi*, and *Cosmopolites sordidus* was found to be 53.9–95.5%, 64.82–81.06%, 74.66–75.67%, 94.05–95.75%, 95%, 76–82%, 86.9–87.3%, 88.1–88.3%, and 77.41–78.19%, respectively [31,32,33,34,35,36,37,38,39,40,45,46,51,101]. The DDA of chitosan of Orthoptera insects like *Calliptamus barbarous*, *Schistocerca gregaria*, *Oedaleus decorus*, *Gryllus bimaculatus*, *Brachystola magna*, *Acheta domesticus*, and *Gryllodes sigillatus* was 70–75%, 98%, 70–75%, 56.47–84.98%, 88.55–91.23%, 62.9–88.5%, and 73.6–81.3%, respectively [32,35,51,54,56,59,103]. The DDA of chitosan of Hymenoptera insects like *Apsis mellifera* and *Vespa orientalis* was as large as 96% [32,65]. The DDA of chitosan of Diptera insects like *Hermetia illucens*, *Chrysomya megacephala*, and Musca domestica was reported to be 66–93%, 89.6%, and 83.8–84.4%, respectively [45,73,75,76,80,83,104]. The DDA of chitosan of Hemiptera insects like Cicada slough was 84.1% [21]. The DDA of chitosan of Dictyoptera insects like *Periplaneta americana*, *Blattella germanica*, and *Eupolyphaga sinensis* was 36.8–90.85%, 31.5%, and 96.09–97.05%, respectively [91,96,103,105]. The DDA of chitosan of *Ephemeroptera* insects like mayflies was 84.3% [100]. A higher DDA indicates a higher proportion of GlcN units, leading to increased positively charged amino groups, which enhances chitosan’s cationic and polycationic properties. Chitosan with a high DDA exhibits enhanced cationic characteristics, making it effective for applications such as antimicrobial agents, drug delivery systems, and flocculants in wastewater treatment. Chitosan with a low DDA is more hydrophobic and can be used for applications in biodegradable films, coatings, and controlled-release systems. 

#### 4.1.3. Molecular Weight 

The molecular weight of chitosan from insects refers to the average size of chitosan molecules obtained from the deacetylation of chitin found in insect exoskeletons. The molecular weight of chitosan is typically expressed in terms of its number-average molecular weight or weight-average molecular weight (Mw). 

The Mw of chitosan from insects is shown in Table 2. Different insect species may have variations in the molecular weight of their chitin, which can affect the resulting molecular weight of chitosan. It was reported that the Mw of chitosan of Lepidoptera insects like silkworms was 40.90 kDa [21]. The Mw of chitosan of Coleoptera insects like *Mealworm*, *Tenebrio molitor*, *Catharsius molossus*, *Leptinotarsa decemlineata*, and *Cosmopolites sordidus* was found to be 39.75 kDa, 812.3 kDa, 450 kDa, 2.676–2.722 kDa, and 343 kDa, respectively [21,31,35,46,51]. The Mw of chitosan of Orthoptera insects like grasshoppers, *Dociostaurus maroccanus*, *Brachystola magna Acheta domesticus*, and *Gryllodes sigillatus* was reported to be 39.89 kDa, 5.6–7.2 kDa, 696.95 kDa, 86.8–344 kDa, and 524 kDa, respectively [21,35,49,56,59]. The Mw of chitosan of Diptera insects like *Hermetia illucens* and *Chrysomya megacephala* was 21–505 kDa and 501 kDa, respectively [80,83,104]. The Mw of chitosan of Hemiptera insects like cicada slough was 37.79 kDa [21]. The Mw of chitosan of Dictyoptera insects like *Periplaneta americana* and *Eupolyphaga sinensis* was 16 kDa and 127.79 kDa, respectively [91,96]. The Mw of chitosan of Ephemeroptera insects like mayflies was found to be 3.69 kDa [100]. The deacetylation process conditions, such as temperature, time, and the concentration of the deacetylating agent, can influence the molecular weight of chitosan. Higher temperatures and longer deacetylation times may lead to lower molecular weights. For example, the Mw of *Acheta domesticus* measured by different studies varies widely from 86.8–344 kDa [56,59]. Various methods have been developed to measure the Mw of chitosan, including gel permeation chromatography, size exclusion chromatography, and viscometry [125,126]. Leke et al. revealed that chitosan samples from black soldier fly (*Hermetia illucens*) meal, extracted by three different sources—enzyme-, microwave-, and ultrasound-assisted methods—displayed both antioxidant and antimicrobial activity. Chitosan Mw had effects on biological activities; high Mw chitosan showed better antimicrobial activity [14]. Controlling and tailoring the Mw of chitosan from insect sources is important for customizing its properties to suit specific applications.

#### 4.1.4. Moisture Content and Ash Content

The measurements of moisture content and ash content provide valuable information about the quality and purity of the chitosan obtained from insects, which are crucial considerations for its various applications. Analytical methods such as gravimetric analysis are commonly employed to determine moisture content and ash content in chitosan samples from insects. The moisture content and ash content of chitin and chitosan from insects are shown in Table 2. The moisture content of chitin from *Ephestia kuehniella* (9.1%), *Tenebrio molitor* (6.2%), *Lucanus cervus* (6.6%), *Polyphylla fullo* (5.9%), *Brachytrupes* (4.00%), *Bradyporus sureyai* (5.2%), *Gryllotalpa gryllotalpa* (6.0%), and *Apsis mellifera* (7.7%) was measured [22,41,49,61,101]. The moisture content of chitosan from *silkworms* (0.07%), *Clanis bilineata* (3.8%), *mealworms* (0.19%), *Tenebrio molitor* (4.2%), *Catharsius molossus.* (6.55), *Calosoma rugosa* (8.8%), *Blaps lethifera* (14.3%), *Pimelia fernandezlopezi* (17.2%), *Cosmopolites sordidus* (2.4%), *grasshoppers* (1.8%), *Brachytrupes* (3.33%), *Apsis mellifera* (17.6%), *Musca domestica* (7.8%), cicada slough (0.18%), and *Eupolyphaga sinensis* (5.19%) was reported [21,25,31,35,45,46,49,101]. Ash content refers to the inorganic residue left behind after chitosan is incinerated at high temperatures. It represents the amount of non-organic or mineral matter present in the chitosan sample. Lower ash content indicates higher purity of the chitosan sample. High ash content may negatively affect the performance of chitosan in specific applications, such as biomedical or food-related uses. The ash content of chitin from *Ephestia kuehniella* (0.14%), *Tenebrio molitor* (3.6%), *Lucanus cervus* (0.6%), *Polyphylla fullo* (1.7%), *Cosmopolites sordidus* (6.4%), *Brachytrupes* (1.00%), *Schistocerca gregaria* (14.1%), *Bradyporus sureyai* (3.8%), *Gryllotalpa Gryllotalpa* (2.1%), and *Apsis mellifera* (2.4%) was tested. The ash content of chitosan from *silkworms* (0.05%), *Clanis bilineata* (0.3%), *mealworms* (0.50%), *Tenebrio molitor* (3.7%), *Catharsius molossus* (0.34%), *Calosoma rugosa* (2.0%), *Blaps lethifera* (1.5%), *Pimelia fernandezlopezi* (2.0%), *Cosmopolites sordidus* (2.2%), *grasshopper* (0.89%), *Brachytrupes* (1.00%), *Schistocerca gregaria* (1.6%), *Apsis mellifera* (9.2%), Musca domestica (8.2%), cicada slough (0.03%), and *Eupolyphaga sinensis* (0.55%) was measured [21,25,31,32,45,46,49,91,101]. By carefully controlling and optimizing moisture and ash content, researchers and industries can produce high-quality chitosan with desired properties, making it suitable for a wide range of applications in areas such as biomedicine, agriculture, and environmental science.

#### 4.1.5. Elemental Analysis

Elemental analysis of chitin and chitosan from insects provides valuable information about the presence and relative abundance of different elements. The percentage of C and N (C%, N%) in chitin and chitosan can vary depending on factors such as the insect species, the extraction process, and the degree of deacetylation. The elemental analysis of chitin from different types of insects, including the carbon, nitrogen, hydrogen, and carbon/nitrogen ratio is shown in Table 3. The percentage of C atoms from chitin and chitosan originating from various insects ranged from 32.09% to 48.90% and 38.98% to 42.76%, respectively. The N value of chitin and chitosan from various insects ranged from 4.63% to 6.9% and 5.93% to 7.3%, respectively. The carbon/nitrogen (C/N) ratio of chitin and chitosan for various insects ranged from 6.13 to 9.49 and 5.35 to 6.57, respectively. For chitosan, the C/N ratio is used to calculate the DDA, which is a measure of the extent of acetyl group removal during the conversion of chitin to chitosan [104,127]. A higher C/N ratio corresponds to a lower DDA, indicating a higher degree of acetylation in the chitosan [128]. Accurately determining the C and N percentages in chitin and chitosan is crucial for quality control, ensuring compliance with industry standards. This information also aids in optimizing extraction and purification processes, producing chitin and chitosan with desired properties for diverse applications.

#### 4.1.6. Solubility

Chitin from insects, and chitin in general, is typically insoluble in water and most common organic solvents, such as ethanol, methanol, acetone, and chloroform, due to many hydrogen bonds and crystalline structure. On the contrary, chitosan, with its partial deacetylation, exhibits improved solubility under acidic conditions [129]. Luo et al. reported that the solubility of chitosan from four different insects, cicada slough, silkworm chrysalis, mealworms, and grasshoppers was 99.3%, 98.7%, 97.4%, 94.3%, respectively [21]. Lucas et al. revealed that the solubility of chitosan from mealworm cuticles was 40.3 ± 0.6% [101]. The solubility of chitosan depends on several factors, including its molecular weight, degree of acetylation, and pH of the solvent. Chitosan with high molecular weight and a high degree of acetylation tend to form aggregates due to intermolecular hydrogen bonding and hydrophobic interactions, leading to decreased solubility [130]. At an acidic pH, chitosan is protonated, and its solubility increases due to electrostatic repulsion between the protonated amino groups. At alkaline pH, chitosan deprotonates, and its solubility decreases due to the formation of insoluble salts [131]. Chitosan’s solubility can be enhanced by modifying its structure, such as by introducing more charged groups, such as carboxyl, hydroxyl, amino, or sulfate groups, into the polymer chain [132]. Additionally, chitosan can be chemically modified to form water-soluble derivatives, such as N-carboxymethyl chitosan or N-succinyl chitosan, which have improved solubility and other properties [133]. For chitin, grafting hydrophilic groups, such as polyethylene glycol or acrylic acid, onto the structure can enhance its solubility in water and polar solvents [134,135]. Understanding the solubility behavior of chitin and chitosan is crucial for designing its applications in various fields, such as drug delivery, wound healing, and tissue engineering [136]. Solubility properties influence the processing and fabrication of chitin and chitosan-based materials, such as films, hydrogels, and nanocomposites [7,137,138].

#### 4.1.7. Fourier Transform Infrared Spectroscopy 

**Figure 4:**
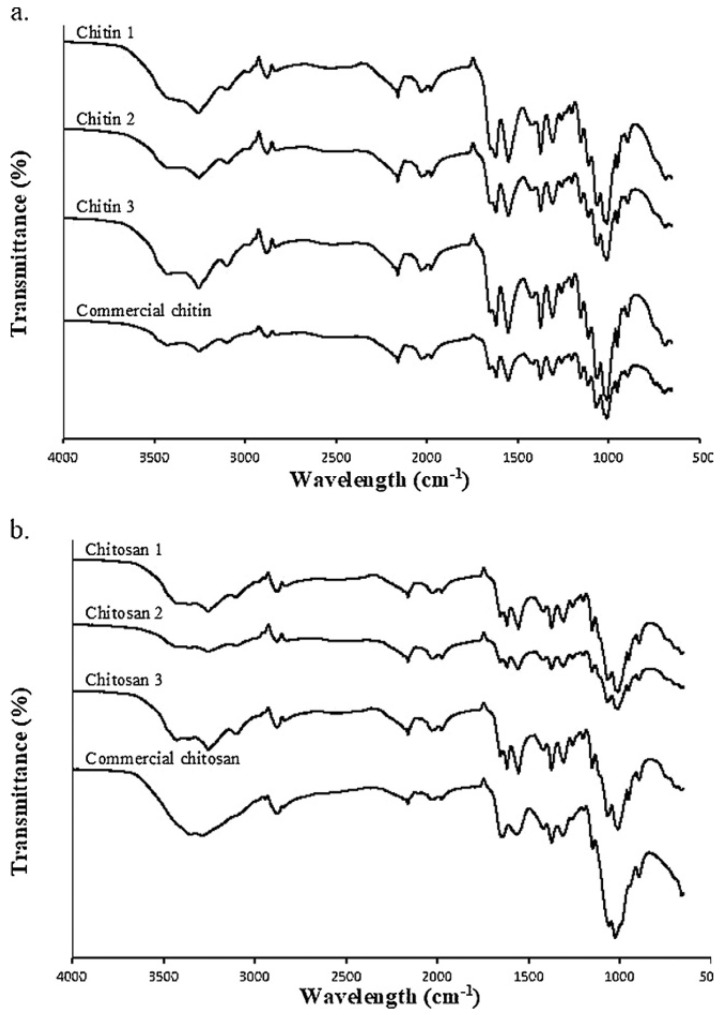
FTIR spectrograms of (**a**) chitin and (**b**) chitosan extracted from *Zophobas morio* larvae in varying sodium hydroxide concentration. Reprinted with permission (5665301211911) from Soon et al. [39].

Fourier Transform Infrared Spectroscopy (FTIR) is a powerful analytical technique used to characterize the molecular structure and functional groups present in chitin and chitosan extracted from insects. Figure 4 presents the classical FTIR spectra of chitin and chitosan extracted from insects. The peak at around 1615–1665 cm^−1^ corresponds to the carbonyl (C=O) stretching vibration of the acetyl (Ac) groups in chitin. It confirms the presence of N-acetylglucosamine units in the chitin structure. The peak at around 1635–1670 cm^−1^ is still present in the chitosan spectrum, but it is usually broader and slightly shifted to lower wavenumbers compared to chitin. This change is due to the partial deacetylation, indicating the presence of both acetyl and amino groups in chitosan. The peak at around 1570–1605 cm^−1^ corresponds to the amide I band, indicating the presence of amide groups (N-H bending) in chitosan. It becomes more prominent with increasing deacetylation, reflecting the higher proportion of glucosamine units. The peak at around 3330–3490 cm^−1^ indicates the presence of hydroxyl (OH) groups in chitin and chitosan, which are involved in hydrogen bonding and contribute to its crystalline structure [37,51,123,124,139]. To differentiate between α-form and β-form chitin content, an additional peak related to the amide I band in the FTIR spectrum can be observed. For example, Soon et al. reported that the chitin isolated from *Zophobas morio* was in α-form because the amide I band was split into two bands, namely, 1620 cm^−1^ and 1650 cm^−1^, respectively [39]. Kaya et al. reported characteristic bands of α-form chitin from *Calliptamus barbarus* (observed at 1652 cm^−1^ and 1621 cm^−1^) and *Oedaleus decorus* (1655 cm^−1^ and 1620 cm^−1^) [51]. Furthermore, the degree of deacetylation in chitosan can be quantified based on the intensity ratio of specific peaks in the FTIR spectrum. One common method is to calculate the DDA as the ratio of the areas under the peaks of acetyl groups and amide groups. A higher DDA value indicates a greater proportion of glucosamine units and a more deacetylated chitosan structure [140].

#### 4.1.8. Crystalline Properties 

**Figure 5:**
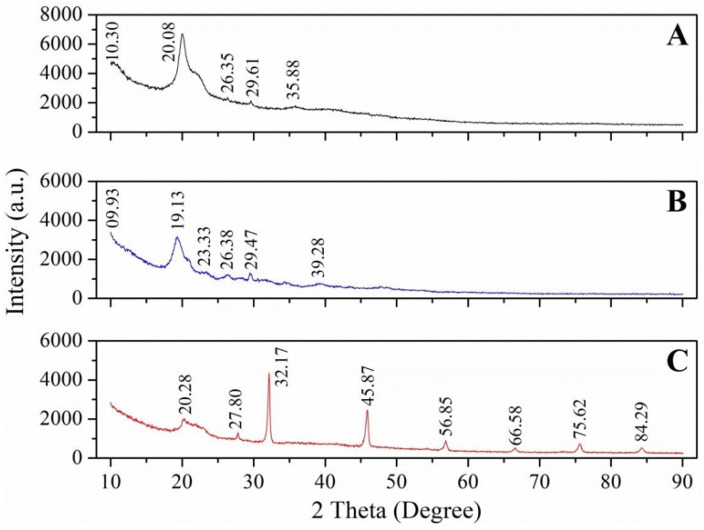
XRD pattern of (**A**) chitin and (**B**) chitosan from cuticles of mealworm (*Tenebrio molitor*), and (**C**) commercial chitosan. Reprinted with permission (5665350347326) from Lucas et al. [101].

The crystalline analysis of insect-extracted chitin and chitosan is vital for understanding their molecular structure and degree of crystallinity, influencing mechanical properties, solubility, and interactions. Common techniques, such as X-ray diffraction (XRD) and solid-state nuclear magnetic resonance, reveal distinct peaks in classical XRD patterns (Figure 5), indicating crystalline regions. Chitin displays well-defined peaks, showcasing a highly ordered structure. Chitosan, with partial deacetylation, may exhibit broader or less intense peaks, signifying a less ordered structure than chitin. The degree of crystallinity, a critical parameter obtained from XRD analysis, quantitatively measures the proportion of crystalline regions compared to amorphous regions. Higher crystallinity suggests a more ordered molecular arrangement, while lower crystallinity indicates a more disordered and amorphous structure. As shown in Table 4, the crystallinity of chitin and chitosan from different insect species ranged from 24.9 to 96.4% and 32.9% to 86.64%, respectively. Wang et al. found that the crystallinity of chitin from *Hermetia illucens* varies greatly at different growth stages, such as larvae (33.09%), prepupa (35.14%), puparium (68.44%), and adult (87.92%) [71]. Kaya et al. also attempted to compare the crystallinity of chitin from different body parts of the Lepidoptera insect *Argynnis pandora*, and the results showed that the crystallinity of wings (64%) was close to other parts (66%) [23]. For the same insect sample, *Hermetia illucens*, the crystallinity of chitin obtained by different methods varied greatly from 24.9% to 94% [67,68,69,71,80]. Crystalline analysis of chitin and chitosan from insect sources is important for understanding the fundamental properties of these biopolymers and for tailoring their characteristics to fit with future applications.

#### 4.1.9. Thermogravimetric Analysis 

**Figure 6:**
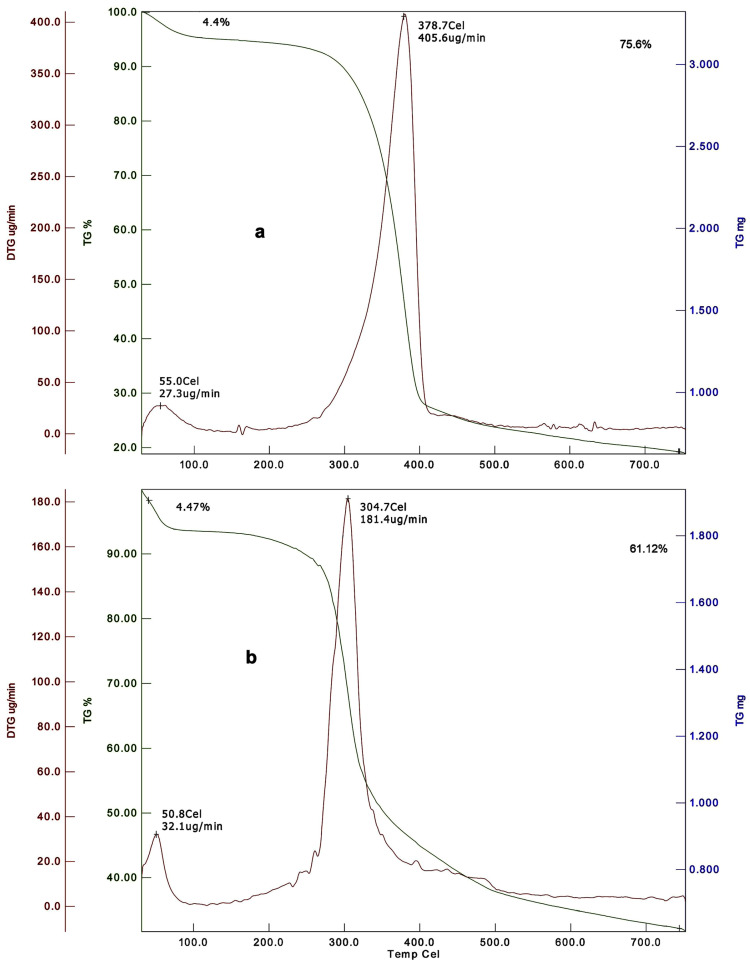
TGA/DTG curves of (**a**) chitin and (**b**) chitosan from *Drosophila melanogaster*. Reprinted with permission (5665311251852) from Kaya et al. [85].

Thermogravimetric analysis (TGA) is a widely used technique to study the weight loss of chitin and chitosan from insects as a function of temperature, and it offers valuable information about their thermal behavior, stability, and decomposition characteristics (Table 5 and Figure 6). The maximum degradation temperature (DTG_max_) represents the temperature at which the sample loses maximum weight due to thermal decomposition. For chitin, the DTG_max_ is usually higher due to its more crystalline and stable structure, while chitosan may have a lower DTG_max_ due to the presence of more amorphous regions [48,51,87,88]. As reported in many papers, the DTG_max_ of chitin and chitosan extracted from different insect orders ranged between 307 and 412.7 °C and 289 and 317.7 °C, respectively. The TGA analysis of chitin and chitosan from various insects reveals that mass loss typically occurred in three stages: the first mass loss occurred around 100 °C linked with water loss, followed by a second and third mass loss (100–750 °C and 200–650 °C), respectively. For all the chitin and chitosan from various insects, the first mass loss was noted to be 1.1–9% and 3–13.37%, respectively, while the second mass loss was 48–94.5% and 45.76–96%, respectively. The TGA curve also shows some residue formation, indicating the presence of inorganic or non-volatile components in the chitin and chitosan samples after thermal decomposition. These results are essential for understanding the thermal characteristics of chitin and chitosan and are valuable for optimizing their processing, quality control, and potential applications in various industries, including biomedicine, agriculture, and environmental science.

#### 4.1.10. Nuclear Magnetic Resonance Spectroscopy 

**Figure 7:**
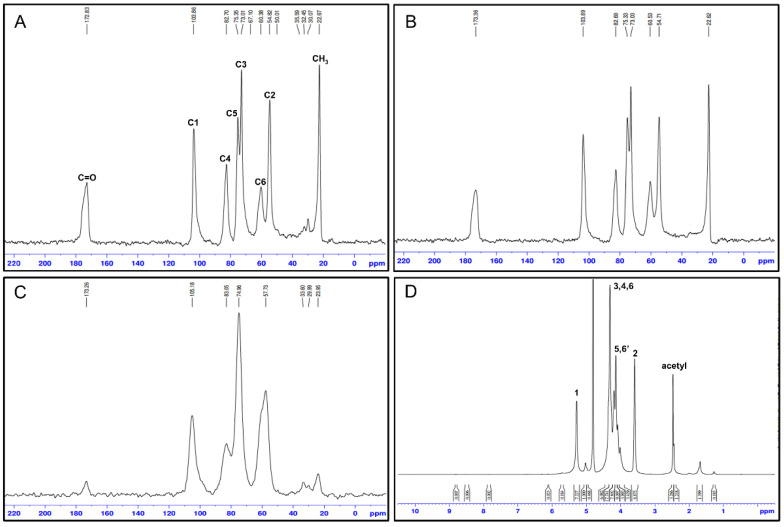
^13^C NMR data of mealworm chitin (**A**), shrimp chitin (**B**), and mealworm chitosan (**C**). ^1^H NMR data of mealworm chitosan (**D**). Reprinted from Son et al. [36].

Analyzing chitin and chitosan from insects using nuclear magnetic resonance (NMR) spectroscopy is a powerful technique that provides detailed structural information about these biopolymers. Solid-state NMR allows for the investigation of materials that do not readily dissolve in traditional liquid-state NMR solvents [28]. As shown in Table 6 and Figure 7, there are some specific peaks and their approximate chemical shifts in the ^13^C NMR spectra of chitin and chitosan [59,64,69,107,108,109]. The acetyl carbon (C=O, CH_3_-CO) in chitin typically appears in the region of 170–175 ppm. Due to the deacetylation process, chitosan may have a peak at a slightly lower chemical shift, around 165–170 ppm, corresponding to the acetyl carbon. The carbon at the anomeric position (C-1) in the glycosidic linkage of both chitin and chitosan typically appears in the range of 100–105 ppm. This carbon is part of the sugar ring structure. C-2, C-3, C-4, and C-5 (carbon atoms in the sugar ring) in the sugar ring structure exhibit chemical shifts in the range of 55–85 ppm, depending on their specific positions within the ring. For example, the peaks of C-2 and C-3 normally appear at 55 ppm and 75 ppm, respectively. Carbon C-6 (carbon in the sugar ring) in the sugar ring typically appears in the range of 55–65 ppm. Chitosan contains hydroxyl groups (OH) introduced during the deacetylation process. The carbons associated with these hydroxyl groups can give rise to peaks in the region of 70–80 ppm, reflecting their different chemical environment. The carbons in the CH_2_ groups within Glucosamine and N-Acetylglucosamine units usually appear in the range of 20–40 ppm. In both chitin and chitosan, carbons associated with the amide linkages can be found in the range of 150–175 ppm. The deacetylation degree of chitin and chitosan could be estimated using ^1^H NMR by comparing signals from acetylated and deacetylated glycosamine units [123,124]. It is important to note that the exact NMR chemical shifts can vary depending on factors such as the source of chitin or chitosan, their degree of deacetylation, and the molecular conformation. 

#### 4.1.11. Scanning Electron Microscopy

**Figure 8:**
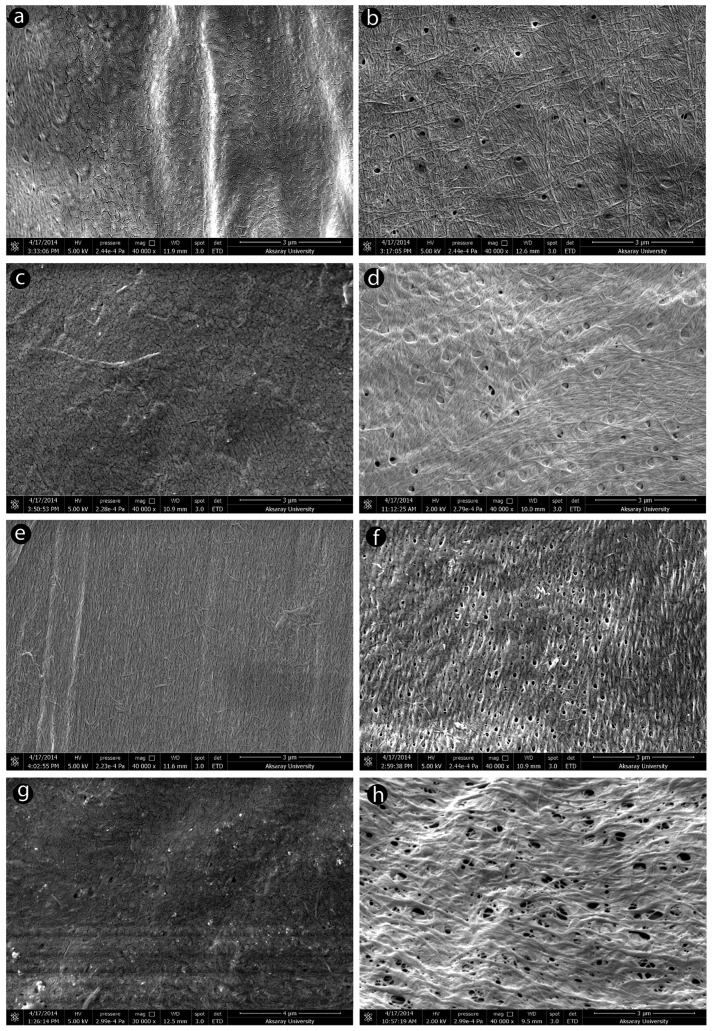
SEM images of four insect species: (**a**) Celes variabilis female, (**b**) C. variabilis male, (**c**) Decticus verrucivorus female, (**d**) D. verrucivorus male, (**e**) Melanogryllus desertus female, (**f**) M. desertus male, (**g**) Paracyptera labiate female, (**h**) P. labiate male. Reprinted from Kaya et al. [50].

The surface morphology of chitin and chitosan, derived from insects and analyzed via Scanning Electron Microscopy (SEM), offers profound insights into their microstructural characteristics. Surface morphologies of insect chitin and chitosan normally showed the following properties: (I) nanofiber, (II) nanopore, (III) smooth surface, and (IV) rough surface, as shown in exemplary Figure 8. Chitin from *Ephestia kuehniella* Zeller, *Vespa crabro*, Vespa orientalis, Vespula germanica, *Blaberus giganteus*, dragonflies (*Sympetrum fonscolombii)*, *Melolontha melolontha*, *Omophlus* sp., *Celes variabilis*, *Decticus verrucivorus*, *Melanogryllus desertus*, *Paracyptera labiate*, *Hermetia illucens*, and *Mecynorhina torquata* exhibit a highly ordered structure composed of microfibers [22,27,28,50,63,68,92,99,123]. These microfibers are the fundamental building blocks of the material and contribute to strength and rigidity. Chitin from *Ephestia kuehniella*, *Vespa crabro*, *Vespa orientalis*, *Vespula germanica*, *Bradyporus* (*Callimenus*) *sureyai*, *Gryllotalpa gryllotalpa*, *Polyphylla fullo*, *Blaberus giganteus*, *Melolontha melolontha*, *Omophlus* sp., and *Mecynorhina torquata* exhibit nanopores that are nano-sized, high-aspect-ratio rods with a distinct surface morphology [22,27,28,41,63,92,123]. These nanostructures are of interest for various applications, including nanocomposites. Kaya et al. compared chitins extracted from male and female *Celes variabilis*, *Decticus verrucivorus*, *Melanogryllus desertus*, and *Paracyptera labiate* using SEM and revealed that male chitin normally shows an obvious nanopore structure [50]. Chitin from different body parts of Argynnis pandora butterflies showed several different types under SEM, such as microfibers, smooth porous zones, plane areas having no pores, and a tough and rough surface [23]. Chitin from Lucanus cervus showed a rough surface [41]. Chitosan, which is derived from chitin through deacetylation, generally has a less crystalline and more amorphous structure. As a result, the surface of chitosan usually appears more irregular and porous under SEM. Luo et al. compared the morphologic properties of chitosan from four insects [21]. The surface structures of cicada slough chitosan were compact and intertwined with each other and showed a needle-like, tighter shape. Silkworm chrysalis chitosan was similar to the reticular structure. Mealworm chitosan has a surface with soft and irregular fibers. Grasshopper chitosan revealed an irregular block and rough structure without any porosity. Marei et al. revealed that the surface of locust chitosan had short, thick, and regularly arranged nanofibers and some pores; beetle chitosan had randomly distributed nanofibers; and honeybee chitosan had a hard and regular rough surface without pores or nanofibers [32]. Kaya et al. revealed that chitin and chitosan from larvae and adult Colorado potato beetles (*Leptinotarsa decemlineata*) have similar nanofiber structures [102]. Ibitoye et al. revealed that chitin and chitosan from house crickets showed similar rough and smooth layers of flakes with big pores and fibers [49]. Chitin and chitosan from *Drosophila melanogaster* also showed similar nanofiber structures [85]. Chitin and chitosan from *Goliathus orientalis* showed similar morphologic properties with a well-defined network and layer-by-layer structure [139]. The above studies indicated that source, sex, body part, and life stage all affected the surface morphology of insect chitin and chitosan. The structures of insect chitin and chitosan have provided sources of inspiration for the fabrication of many novel materials for various biomimetic applications, such as nanocomposites.

## 5. Biomedical Applications of Chitin and Chitosan from Insects

**Figure 9:**
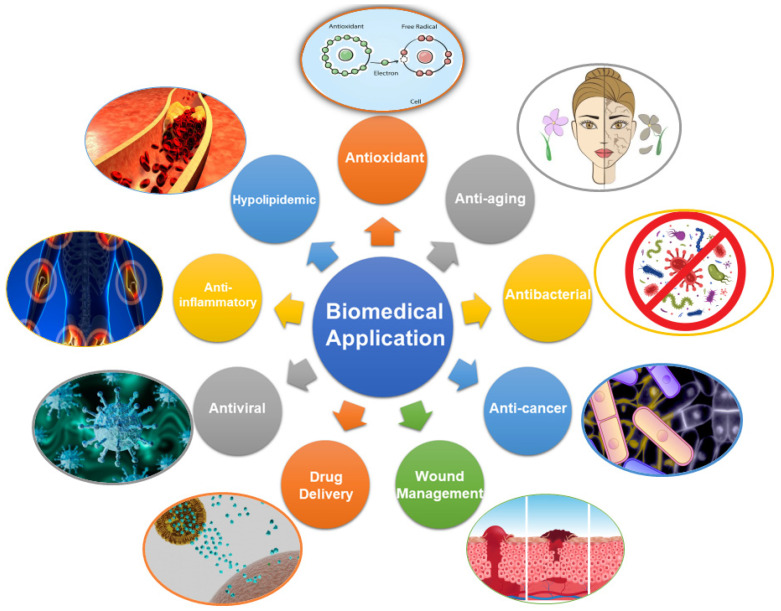
Biomedical applications of chitin and chitosan from insects.

Biomimetic chitosan-based materials with tunable morphological, biological, and physicochemical features have been used in various fields. Chitin and chitosan offer a wealth of promising biomimetic applications owing to their unique biocompatibility, biodegradability, and versatile chemical properties, inspiring innovative solutions in drug delivery, wound healing, antioxidants, and more (Figure 9). 

### 5.1. Antioxidant and Anti-Agng Activity

Free radicals can damage cells, proteins, and DNA, contributing to various health issues and accelerating the aging process. Antioxidants work by neutralizing free radicals, thus reducing the risk of cellular damage and chronic diseases [141]. Anti-aging approaches focus on promoting skin health, maintaining cognitive function, preventing chronic diseases, and enhancing overall quality of life [142]. The relationship between antioxidants and anti-aging is rooted in the idea that antioxidants can help to protect the body from the damage caused by oxidative stress, potentially slowing down the aging process and promoting longevity [143]. Many studies have reported the antioxidant and anti-aging activities of insect chitosan-based biomimetic materials. Chitosan film from the American cockroach *(Periplaneta americana*) could resist UV light effectively and keep scavenging DPPH radicals for 8 h [96]. Wu et al. found that the chitosan of *Clanis bilineata* larva skin exhibited high antioxidant activity in vitro and anti-aging activities in D-gal-induced mice [26]. The antioxidant capacities of the chitosan from two gregarious Orthoptera species (*Calliptamus barbarus* and *Oedaleus decorus*) were evaluated using the free radical scavenging activity (2,2-diphenyl-1-picrylhydrazyl, DPPH) and ferric reducing power assays, and the scavenging effect increased with increasing concentrations of the extracts [51]. Chu et al. found that both DPPH radical scavenging and ferric-chelating assays showed a positive correlation with the deacetylation degree of chitosan isolated from superworm (*Zophobas morio*) larvae [39]. Tafi et al. reported that chitosan from *Hermetia illucens* as a coating for the preservation of a fresh food product was generally able to increase the phenolic and flavonoid content and the antioxidant activity of tomatoes during both RT and cold storage [144].

### 5.2. Antibacterial Activity 

Chitosan has gained significant attention for its antibacterial properties, which are attributed to its unique chemical structure and versatile applications (Table 7). Kaya et al. reported that the antimicrobial activities of the chitosan (250 μg/disc) from two gregarious Orthoptera species (*Calliptamus barbarus* and *Oedaleus decorus*) were more effective than traditional antibiotics (Amikacin, Ampicillin, Gentamicin, Erythromycin, and Kanamycin) against some of the pathogenic bacteria strains studied. The chitosan from these two species generally showed stronger antibacterial effectiveness against the Gram-negative bacteria than the Gram-positive bacteria [51]. The negative charge on the cell surface of the Gram-negative bacteria is higher than that on the Gram-positive bacteria, which causes a greater amount of chitosan to be adsorbed and a higher inhibitory effect against the Gram-negative bacteria [145]. Mahboub et al. reported that chitosan nanoparticles prepared from the American cockroach, *Periplaneta americana*, inhibit the growth of *Escherichia coli*, *Klebsiella pneumoniae*, *Staphylococcus aureus*, and *Bacillus subtilis.* Chitosan nanoparticles were found to cause the deformation and rupture of the selected bacteria by transmission electron microscopy (TEM) investigation [97]. The chitosan from mealworm beetles (*Tenebrio molitor*, *Zophobas morio*) showed an antibacterial effect against *Pseudomonas aeruginosa* [37]. Shin et al. also confirmed that *Tenebrio Molitor* chitosan had about 1–2 mm inhibition zones against four strains of bacteria: *Bacillus cereus*, *Listeria monocytogenes*, *Escherischia coli*, and *Staphylococcus aureus*, indicating antimicrobial activity [38]. Chitosan extracted from *Blaps lethifera*, *Pimelia fernandezlopezi*, and *Musca domestica*, showed good antibacterial activity against *Listeria innocua*, *Bacillus subtiliis*, *Staphylococcus aureus*, *Salmonella typhimurium*, and *Pseudomonas aeruginosa* [45]. 

Many researchers have been focusing on chitosan from the black soldier fly (BSF), *Hermetia illucens*. Teo et al. found that BSF chitosan could restrict bacterial growth at concentrations of 0.25% or 0.5%, with the two most susceptible species being identified as *Pseudomonas aeruginosa* and *Serratia marcescens* [75]. Lagat also revealed that BSF chitosan concentrations of 2.5 and 5 g/mL significantly inhibited the growth of *Escherichia coli*, *Bacillus subtilis*, *Pseudomonas aeruginosa*, *Staphylococcus aureus*, and *Candida albicans* [76]. Anna et al. revealed that chitosan from different biomasses (larvae, pupal exuviae, and dead adults) of BSF had good antimicrobial ability both against Gram-negative and Gram-positive bacteria species [77]. Because of antibacterial activity, insect chitosan has been considered for use in the textile field and food packaging. Parag et al. used BSF chitosan as a finishing agent for polyester fabrics using citric acid as a cross-linking agent [146]. 

Biomimetic chitosan-based film from two reared cricket species (*Acheta domesticus* and *Gryllodes sigillatus*) showed the potential for use as bio-based packaging material for food and pharmaceutical applications because of good mechanical and barrier properties and improved water resistance and light barrier characteristics [57]. 

Malm et al. have confirmed that chitosan from *Acheta domesticus* and *Gryllodes sigillatus* effectively inhibited *Escherichia coli* and *Listeria innocua*. Also, their antimicrobial activity against *Escherichia coli* was more effective at higher DDA values compared to shrimp chitosan [59]. Chitosan from three species of American cockroach, *Periplaneta american*; the German cockroach, *Blattella germanica*; and the mealworm beetle, *Tenebrio molitor* were investigated for their anti-bacterial properties. The results showed that the anti-bacterial influence of the chitosan is based on the insect species and chitosan concentration, and American cockroach chitosan at a concentration of 1% had the greatest effect on *Proteus mirabilis*, while German cockroach chitosan at a concentration of 0.01% had the greatest effect on *Klebsiella pneumoniae* [105]. Hamidreza et al. also confirmed that chitosan from adult and nymph American cockroaches, *Periplaneta american*, and German cockroaches, *Blattella germanica*, had antibacterial and antifungal activities [93]. Chen et al. reported that the chitosan film from *Periplaneta americana* resisted the growth of *Serratia marcescens* and *Escherichia coli* more effectively than shrimp chitosan film [96]. Khayrova et al. found that the chitosan’s source (*Hermetia illucens* larvae or commercial crab) and depolymerisation enzymes (*Myceliophthora thermophila*, *Trichoderma harzianum*, and *M. thermophila*) had significant influence on antibacterial and anti-fungal activities of the obtained low molecular weight chitosan [83]. Chitosan from *Hylobius abietis* was reported to have antimicrobial activity against 18 bacterial strains [147]. Nanoparticles based on Egyptian wasp (*Vespa orientalis*) chitosan (WCSNPs) showed antibacterial activities against extended-spectrum beta-lactamase (ESBL)- and carbapenemase-producing *Klebsiella pneumoniae*, *Escherichia coli*, and *Pseudomonas aeruginosa*. The zeta potential indicated stable WCSNPs capable of binding to the cellular membrane and increasing cellular uptake [65]. Chitosan from *Cosmopolites sordidus* revealed concentration-dependent antibacterial activity against *Escherichia coli* and *Klebsiella pneumoniae*, and followed with mean growth inhibition zones of 5 mm (3 mg/mL) and 7 mm (4 mg/mL) [46]. 

**Table 7:** Antibacterial activity of insect chitin and chitosan.

Insect Order	Insect Species	Bacteria Strains	Ref.
Coleoptera	*Cosmopolites sordidus*	*Escherichia coli*, *Klebsiella pneumoniae*	[46]
	*Tenebrio molitor*	*Proteus mirabilis*, *Klebsiella pneumoniae*, *Enterococcus faecalis*, *Staphylococcus epidermidis*	[105]
	*Tenebrio Molitor*	*Bacillus cereus*, *Listeria monocytogenes*, *Escherischia coli*, *Staphylococcus aureus*	[38]
	*Hylobius abietis* L.	*Enterococcus faecalis*, *Enterobacter aerogenes*, *Bacillus subtilis*, *Streptococcus pneumonia*, *Vibrio parahoe molyticus*, *Acinetobacter baumannii*, *Bacillus megaterium*, *Bacillus cereus*, *Micrococcus luteus* *Klebsiella oxytoca*, *Pseudomonas aeruginosa*, *Shigella sonnei*, *Staphylococcus epidermitis wt*, *Staphylococcus aureus*, *Klebsiella pneumoniae subsp. Pneumoniae*, *Pseudomonas aeruginosa*, *Pseudomonas aeruginosa*, *Escherichia coli*	[147]
	*Tenebrio molitor*	*Pseudomonas aeruginosa*	[37]
	*Blaps lethifera*, *Pimelia fernandezlopezi*	*Listeria innocua*, *Bacillus subtiliis*, *Staphylococcus aureus*, *Salmonella typhimurium*, *Pseudomonas aeruginosa*	[45]
	*Tenebrio molitor*	*Proteus mirabilis*, *Klebsiella pneumoniae*, *Enterococcus faecalis*, *Staphylococcus epidermidis*	[105]
Dictyoptera	American Cockroach German Cockroach	*Micrococcus luteus*, *Staphylococcus aureus*, *Pseudomonas aeruginosa*, *Escherichia coli*	[93]
	American cockroach, *Periplaneta americana*	*Escherichia coli*, *Klebsiella pneumoniae*, *Staphylococcus aureus*, *Bacillus subtilis*	[97]
	*Periplaneta americana*	*Serratia marcescens*, *Escherichia coli*	[96]
	*Periplaneta americana* *Blattella germanica*	*Proteus mirabilis*, *Klebsiella pneumoniae*, *Enterococcus faecalis*, *Staphylococcus epidermidis*	[105]
Diptera	black soldier fly, *Hermetia illucens*	*Pseudomonas aeruginosa*, *Serratia marcescens*	[75]
		*Escherichia coli*, *Bacillus subtilis*, *Pseudomonas aeruginosa*, *Staphylococcus aureus*, *Candida albicans*	[76]
		*Escherichia coli*, *Micrococcus favus*	[77]
		*Escherichia coli*, *Staphylococcus epidermidis*	[83]
	*Musca domestica*	*Listeria innocua*, *Bacillus subtiliis*, *Staphylococcus aureus*, *Salmonella typhimurium*, *Pseudomonas aeruginosa*	[45]
Orthoptera	*Calliptamus barbarous*, *Oedaleus decorus*	*Listeria monocytogenes*, *Bacillus subtilis*, *Salmonella enteritidis*, *Yersinia enterocolitica*, *Candida albicans*	[51]
	*Acheta domesticus*, *Gryllodes sigillatus*	*Escherichia coli*, *Listeria innocua*	[59]
Hymenoptera	*Vespa orientalis*	*Klebsiella pneumoniae*, *Escherichia coli*, *Pseudomonas aeruginosa*	[65]

### 5.3. Anticancer Activity

The anticancer activity of insect chitosan has been demonstrated in many studies. Hasaballah et al. investigated the anticancer activity of chitosan nanoparticles (CNPs) from maggots of *Musca domestica*, *Lucilia sericata*, and *Chrysomya albiceps* against human liver carcinoma (HepG-2) and human colon carcinoma (HCT-116) cell lines. Their anticancer activities in both tested cell lines were found to be highly effective and concentration-dependent; the highest anticancer activity was recorded at concentrations of 80, 90, and 100 μg/mL, and median inhibitory concentration (IC50) values were in the range of 37.3 to 74.3 μg/mL. Tan et al. revealed that the effects of chitosan vary depending on cell types, concentration, and chitosan derivatives. At concentrations below 250 μg/mL, mayfly and commercial chitosan with low and medium Mw exhibited strong inhibitory activity on cancer cells (A549 and WiDr cells). Mayfly chitosan induced early and late apoptosis in A549 cells, but late apoptosis and necrosis in WiDr cells [100]. Mahboub et al. investigated the anticancer activity of chitosan prepared from the American cockroach against Hepatoblastoma (HepG2) and breast cancer (MCF7) cell lines with the MTT assay. The cytotoxicity was found to have a positive relationship with the chitosan concentration, and the IC50 was 329 and 195 μg/mL with HepG2 and MCF7, respectively [148].

### 5.4. Insect Chitosan-Based Biomimetic Materials for Wound Management

Chitosan from different natural sources has been widely utilized in various forms, including wound dressings, gels, films, and sponges, to support the wound healing process due to its unique properties of hemostatic, anti-inflammatory, antimicrobial, biocompatibility, biodegradability, and promotion of granulation tissue formation [149]. The wound-healing activity of insect chitosan-based biomimetic nanofibers has been reported in some studies. Jiang et al. mixed chitosan from *Eupolyphaga sinensis* Walker with PVA and PEO (DCS/PVA/PEO) to create a novel electro-spun nanofiber membrane, whose activities of accelerating wound healing and collagen regeneration were verified with a rat full-thickness skin defect model. The structure of DCS/PVA/PEO, like the extracellular matrix, provides a suitable environment for soft tissue regeneration, which could promote cell growth. It could be used as a carrier for delivering various drugs and active substances or to develop new wound dressings [91]. Marei et al. confirmed the biocompatibility and function of accelerating wound healing of chitosan from desert locusts (*Schistocerca gregaria*) with in vitro and in vivo models. Compared to shrimp chitosan, insect chitosan showed earlier granulation as well as dermis-active angiogenesis, with a significantly higher count with early marked epithelization and formation of a thicker epidermis with minimal inflammation [150]. 

### 5.5. Insect Chitosan-Based Biomimetics Drug Delivery System

Chitosan from different sources has been engineered into different types of biomimetic drug delivery carriers, such as nanoparticles, microspheres, membranes, sponges, and rods, and has been used in the design of many various administration routes such as oral, buccal, nasal, transdermal, parenteral, vaginal, cervical, intrauterine, and rectal [151,152,153]. For the drug delivery activity of insect chitosan, some studies have been reported. Magdalena et al. reported that chitosan from crickets was used with alginate for the preparation of polymer capsules, which can be applied for the delivery of nisin. Compared to alginate-based capsules, chitosan–alginate-based capsules had a two times longer period of release, indicating that the possibility of achieving an extended-release profile of an active substance is a big advantage of the developed systems. Due to the electrostatic interactions between OH^−^ and COO^−^ ions, the release was significantly more effective in alkaline solutions [154]. Sedef et al. revealed that quercetin (80 μg/mL)-loaded chitosan membranes from *Tabanus bovinus* corneal lenses presented antibacterial effectivity, as much as commercial gentamicin antibiotics. Quercetin-loaded chitosan membranes had a higher antimicrobial effect against Gram-negative bacteria (*Escherichia coli*) than against Gram-positive bacteria (*Staphylococcus aureus*). They have been shown to work with nanoparticles and are adoptable for the release of nano-based fluidic systems for various biomedical applications [87]. Marei et al. prepared nanoparticles loaded with ciprofloxacin (Cipro/CSNPs) with chitosan isolated from desert locusts, beetles, honeybee exoskeletons, and shrimp shells, which could inhibit the growth of *Escherichia coli*, *Bacillus thuringiensis*, Methicillin-resistant *Staphylococcus aureus* (MRSA), and *Pseudomonas aeruginosa*, and showed higher antibacterial activity than nanoparticles or chitosan itself. CSNPs had the highest antibacterial activity against E. coli and MRSA, with MIC varying from 0.0043 to 0.01 μg/mL and from 0.07 to 0.14 μg/mL, respectively. The locust chitosan-based nanoparticles had the smallest particle size, 36.7 ± 3.59 nm, which increased the drug penetration into the Escherichia coli bacterial cell and improved antibacterial activity after drug loading. The minimum inhibitory concentration (MIC) value was 85.6% lower than the MIC of the free drug itself [33]. It was predicted that biomaterials based on insect chitosan could be used in medicine as a carrier for various agents for their biocompatibilities.

### 5.6. Other Biomedical Applications 

In addition to those listed above, insect chitosan has other biological activities, such as antiviral, anti-inflammatory, hypolipidemic, and so on. Mahboub et al. reported that novel biomimetic chitosan-based nanoparticles (CNPs) from American cockroaches (*Periplaneta americana*) protected the Vero cells from the cytopathic effect of adeno-40 and coxsackie B4 viruses. At the concentration of (80 μg/mL), CNPs substantially reduced adenovirus infectivity titer to 4.2 log (10)/0.1 mL (*p* < 0.05) and coxsackie B4 virus infectivity titer to 4.4 log (10)/0.1 mL (*p* < 0.01). The reduction percentages were about 5 and 26 for adeno-40 and coxsackie B4 viruses, respectively [30]. Son et al. revealed that chitosan from mealworms (*Tenebrio molitor*) had a notable NO reduction effect in the LPS-induced RAW 264.7 cell line assay, and its efficacy was relatively higher than chitosan obtained from other animal sources [36]. Malm et al. revealed that chitosan from the house cricket (*Acheta domesticus*) and tropical banded cricket (*Gryllodes sigillatus*) both showed similar (*p* > 0.05) lipid-binding capacity to that of shrimp chitosan. Varying the DDA had no effect on chitosan’s lipid-binding capacity [59]. Insect chitosan biocompatibility, biodegradability, and various biological activity, combined with its ability to support tissue regeneration and controlled drug release, make it a valuable material in a range of biomedical applications. Its versatility continues to drive innovation in the field of healthcare and biotechnology.

## 6. Conclusion and Future Perspectives

In this review of insect-derived chitin and chitosan, from the nuanced exploration of their sources and production of diverse biomedical applications, these biopolymers stand as remarkable subjects of study with implications that extend into numerous domains, particularly in the realm of biomimetics. The use of insects as a source of chitin and chitosan opens up exciting possibilities due to their abundant availability, rapid growth, and high chitin content. Insects, being one of the most diverse and abundant groups of organisms on the planet, possess exoskeletons rich in chitin. While chitin and chitosan have been extensively studied and applied in various fields, the utilization of insects as a source of chitin and chitosan for biomedical application is relatively new and explored incompletely. The adoption of chitin and chitosan derived from insects contributes to the conservation of natural resources by utilizing insect waste as a valuable and renewable source, supporting resource conservation and reducing the reliance on non-renewable resources. Meanwhile, production techniques of chitin and chitosan from insects have advanced toward sustainability and have become greener, pioneering methods such as fermentation, deep eutectic solvents, or microwave extraction, which reduce energy consumption and environmental impact. Various advanced characterization techniques have empowered the fine-tuning of biopolymer properties to meet specific application demands. For example, Chitosan with a lower Mw tends to have better solubility in water and other solvents, making it more suitable for certain applications like film-forming and solution-based formulations [11]. Higher Mw chitosan is often associated with improved mechanical strength, making it more suitable for applications requiring structural integrity, such as biomedical scaffolds [155]. The ability to customize chitin and chitosan properties for specific applications holds tremendous promise for biomimetic innovation.

Although insect-derived chitin and chitosan have potential commercial medical applications, the applications and product development in the marketplace are still in the research stage, and there are no mature products for medical use. Very limited information on the commercial use of insect-derived chitin and chitosan can be searched. The company, Sfly^®^, utilizes *Hermetia illucens* larvae for high-quality chitin/chitosan production. Stable models for large-scale production, including insect species selection and purification methods, should be further explored. Drawing inspiration from nature’s design principles by mimicking the adaptability, resilience, and biocompatibility of natural materials like insect chitin and chitosan, the potential of these biopolymers extends into diverse domains. In addition, to accommodate the continuous deepening and expansion of insects’ chitin and chitosan in biomedical applications, regulatory standards need to be evolved to ensure safety and efficacy.

## Abbreviations

DDA, degree of deacetylation; DES, deep eutectic solvents; NMR, nuclear magnetic resonance; FTIR, Fourier Transform Infrared Spectroscopy; Mw, molecular weight; DTG, Derivative thermogravimetry; TGA, Thermogravimetric analysis; XRD, X-ray diffraction; SEM, Scanning Electron Microscopy; DPPH, 2,2-diphenyl-1-picrylhydrazyl; BSF, black soldier fly; MIC, Minimum inhibitory concentration; CNPs chitosan nanoparticles.
